# Supplementary material for: Association between patient activation, self-management behaviours and clinical outcomes in adults with diabetes or related metabolic disorders: a systematic review and meta-analysis protocol
Source: BMJ Open. 2022 Jan 31;12(1):e056293. doi: 10.1136/bmjopen-2021-056293 (PMC8804633; doi:10.1136/bmjopen-2021-056293)
Supplement: Supplementary data [file bmjopen-2021-056293supp001.pdf]

Appendix A: Example search strategy

Medline (Ovid)

1	("patient* activation*" or (measure* adj5 "patient activation") or PAM?22* or PAM?13* or PAM??13* or PAM??22* or "Patient Assessment of Chronic Illness Care*" or PACIC*).mp. [mp=title, abstract, original title, name of substance word, subject heading word, floating sub-heading word, keyword heading word, organism supplementary concept word, protocol supplementary concept word, rare disease supplementary concept word, unique identifier, synonyms]
2	(Diabet* or T2DM or T1DM or (non insulin* depend* or non insulin depend* or non insulin?depend* or non insulin?depend) or IDDM or NIDDM or MODY or T1D or T2D).mp. or exp Diabetes Mellitus, Type 2/ or exp Diabetes Mellitus/ or exp Diabetes Mellitus, Type 1/ or exp diabetes insipidus/ or exp Diabetes, Gestational/ [mp=title, abstract, original title, name of substance word, subject heading word, floating sub-heading word, keyword heading word, organism supplementary concept word, protocol supplementary concept word, rare disease supplementary concept word, unique identifier, synonyms]
3	(Pre?diabet* or (Borderline adj3 diabet*) or (Impair* adj3 glucose) or (Non-diabetic adj3 hyperglyc?emi*) or (Glucose adj3 intoleran*)).mp. or exp Prediabetic State/ or exp Glucose Intolerance/ [mp=title, abstract, original title, name of substance word, subject heading word, floating sub-heading word, keyword heading word, organism supplementary concept word, protocol supplementary concept word, rare disease supplementary concept word, unique identifier, synonyms]
4	(Obes* or Overweight or "over weight" or (Body adj3 weight) or "body weight" or Adiposit* or (Weight adj3 (gain* or loss* or chang* or control* or maintain* or reduc* or manag*)) or Bmi or body mass ind*).mp. or exp Obesity/ or exp Overweight/ or exp Body Weight/ or exp Adiposity/ or exp body mass index/ [mp=title, abstract, original title, name of substance word, subject heading word, floating sub-heading word, keyword heading word, organism supplementary concept word, protocol supplementary concept word, rare disease supplementary concept word, unique identifier, synonyms]
5	(Heart* or cardiovascular or coronary or cardio* or cardiac*).mp. or exp Heart Diseases/ or exp Cardiovascular Diseases/ or exp Coronary Disease/ or exp Heart Failure/ [mp=title, abstract, original title, name of substance word, subject heading word, floating sub-heading word, keyword heading word, organism supplementary concept word, protocol supplementary concept word, rare disease supplementary concept word, unique identifier, synonyms]
6	2 or 3 or 4 or 5
7	1 and 6

Mueller J, *et al. BMJ Open* 2022; 12:e056293. doi: 10.1136/bmjopen-2021-056293

Appendix B: Data extraction sheet

Section 1: General meta-data

**Review title**	The association between patient activation, self-management behaviours and clinical outcomes in diabetes and related metabolic disorders: A systematic review						
**Study ID** *(surname of first author and year first full report of study was published e.g. Smith 2001)*							
**Date form completed** *(dd/mm/yyyy)*							
**Initials of person extracting data:**							
**Title:**							
**Author(s):**							
**Source:**							
**Date:**		**Vol:**		**Issue:**		**Pages:**	
**Publication type** (e.g. full report, abstract)							

Section 2: Study eligibility

Study characteristics	Eligibility criteria	Eligibility criteria met?		
		Yes	No	Un-clear
Population	Adults (≥ 18 years old) with diabetes or a related metabolic disorder (prediabetes, type 1 and type 2 diabetes , obesity, or CVD)	☐	☐	☐
Exposure	Includes a measure of patient activation (PAct)	☐	☐	☐
Outcomes	Includes at least one of the predefined outcomes, either clinical outcomes (HbA1C level/ glycaemic control, systolic blood pressure, diastolic blood pressure, low-density lipoprotein (LDL), high-density lipoprotein (HDL), total cholesterol, serum	☐	☐	☐

	triglycerides, BMI / weight, life expectancy/survival) or self-management behaviours (diet, physical activity, smoking, alcohol, medication adherence)			
Type of study	Original, primary research articles	☐	☐	☐
	Assesses the relationship between PAct and at least one of the defined outcomes	☐	☐	☐
	If no to the above: Is it an intervention study that reports intervention effects on PAct and effects on other specified outcomes AND (i) the intervention explicitly aims to increase patient activation or is described as targeting patients' knowledge, confidence and skills for self-management AND (ii) increasing patient activation is the main component of the intervention	☐	☐	☐
INCLUDE ☐		EXCLUDE ☐		
Reason for exclusion:				

DO NOT PROCEED IF STUDY EXCLUDED FROM REVIEW

Section 3: Objectives and design

**Objective:**	
**Setting:**	
**Country of origin:**	
**Start and end date:**	
**Study design**	
**Study population:**	
**Recruitment methods:**	
**Inclusion and exclusion criteria for participants:**	
**Sample size:**	

Mueller J, *et al. BMJ Open* 2022; 12:e056293. doi: 10.1136/bmjopen-2021-056293

**Is a justification for the sample size provided (power calculation)?**	Yes/No (delete as appropriate)<br><br>Details:
**Withdrawals and exclusions:**	
**Attrition (i.e. loss to follow-up):**<br><br>**(For intervention studies, report per study group)**	

Section 2: Intervention details

Only complete Section 2 if it is an intervention study and we are interested in findings that depend on study group allocation. If it is an observational study, or an intervention study but the relevant data to extract pertain to the association between PAct and outcomes independent of study group allocation, skip to section 3.

Mueller J, *et al. BMJ Open* 2022; 12:e056293. doi: 10.1136/bmjopen-2021-056293

	**Descriptions as stated in the report/paper**
**Randomisation and blinding:**	
**Sample size per group**	Intervention:<br>Control:
**Any indication for baseline differences between study groups?**	Yes/No/Unclear<br>Details:
**Comparison group description**	
**Intervention aim**	
**Is the explicit main aim of the intervention to increase patient activation or to target patients' knowledge, confidence and skills for self-management?**	*Yes/No/Unclear*<br>*(Delete as appropriate. Select No if the patient activation component forms part of a larger complex intervention).*
**Is patient activation the main component of the intervention?**	
**Intervention description**	
**Group or individual delivery**	
**Mode of delivery (e.g. web, face-to-face)**	
**Duration of intervention**	
**Timing (e.g. frequency, duration of each session)**	
**Providers (e.g. profession and training received)**	
**Intention to treat analysis?**	*Yes/No/Unclear (Delete as appropriate).*
**Any further notes:**	

Section 3: Outcomes & Measures

**PAct measure**	
**PAct measure used as continuous measure, ordinal (levels 1-4), or dichotomous (high/low)?**	Continuous/ordinal/dichotomous<br>(delete as appropriate)
**Time points measured/reported (for all outcomes):**	

**Covariates:**<br>*(Note: Only extract covariates that were included in models that assessed the association between PAct and the outcomes of interest as per review protocol)*	

Clinical outcomes

Note: If outcomes not measured, please insert "n/a"

	**How measured/defined (+unit of measurement)**	**Source (e.g. self-report, health records)**
HbA1C level/glycaemic control		
Systolic blood pressure, diastolic blood pressure		
Low-density lipoprotein (LDL)<br>High-density lipoprotein (HDL)<br>Total cholesterol		
Serum triglycerides		
BMI		
weight		
Life expectancy/survival		

Self-management behaviours

Note: If outcomes not measured, please insert "n/a"

	**Self-report? (Yes/No/Unclear)**	**How defined/measured?** ***e.g. "consuming 5 servings of fruit/veg per day (Yes/No)"***
Diet		
Physical activity		
Smoking		
Alcohol consumption		
Medication adherence		

Section 4: Analyses + Results

Please extract data for adjusted and unadjusted associations (i.e. associations just between PAct and the relevant outcome [=unadjusted], and those where a model such as a linear regression is used to control for confounders [=adjusted]). If extracting data for both adjusted and unadjusted associations, please add additional rows to the table (e.g. an additional row labelled 'Cross-sectional association with PAct' so that you have one for the adjusted and one for the unadjusted data).

If several time points are reported, extract data for the longest follow-up time point.

If several variables were used for the same outcome please copy and paste the table and add details for the respective variable (for example, create a second table for "diet", and add the variable.

If the format of the tables is unsuitable for the reported results, please paste the relevant results into the 'other/comments' section.

How were missing data handled? *(e.g. multiple imputation)*	

**Outcome: HbA1c/glycaemic control**						
**How measured/defined:**						
	**Statistical test**	**Adjusted/ unadjusted?**	**Covariates (if adjusted)**	**Effect size for the association** (e.g. χ2, F, t or p values, Odds ratios, beta coefficients)	**p**	**Sample size**
Cross-sectional association with PAct:						
If intervention/longitudinal: Association between baseline PAct and subsequent outcome:						
If intervention/longitudinal: Association between baseline PAct and change in outcome:						
If intervention/longitudinal:						

Association between change in PAct and subsequent outcome:						
If intervention/longitudinal: Association between change in PAct and change in outcome:						
Other/comments:						

To extract data for further outcomes, please copy and paste the table above and edit the "outcome" field.

Outcomes:

- systolic blood pressure
- diastolic blood pressure
- LDL/HDL/Total cholesterol
- serum triglycerides
- weight
- BMI
- Life expectancy/survival
- Diet
- Physical activity
- Smoking
- Alcohol
- Medication adherence

Mediation:

Only if intervention study. Add details of any formal mediation analyses to determine if PAct mediates intervention effects on outcomes.

Section 5: Conclusions

**Conclusions**	
**Author's conclusions:**	

**Limitations (e.g. multiplicity)**	
**Reviewer's conclusions/comments:**	
