# Supplementary material for: Association between patient activation, self-management behaviours and clinical outcomes in adults with diabetes or related metabolic disorders: a systematic review and meta-analysis protocol
Source: BMJ Open. 2022 Jan 31;12(1):e056293. doi: 10.1136/bmjopen-2021-056293 (PMC8804633; doi:10.1136/bmjopen-2021-056293)
Supplement: Author's manuscript [file bmjopen-2021-056293.draft_revisions.pdf]

BMJ Open

BMJ Open is committed to open peer review. As part of this commitment we make the peer review history of every article we publish publicly available.

When an article is published we post the peer reviewers' comments and the authors' responses online. We also post the versions of the paper that were used during peer review. These are the versions that the peer review comments apply to.

The versions of the paper that follow are the versions that were submitted during the peer review process. They are not the versions of record or the final published versions. They should not be cited or distributed as the published version of this manuscript.

BMJ Open is an open access journal and the full, final, typeset and author-corrected version of record of the manuscript is available on our site with no access controls, subscription charges or pay-per-view fees (http://bmjopen.bmj.com).

If you have any questions on BMJ Open's open peer review process please email info.bmjopen@bmj.com

BMJ Open

The association between patient activation, self-management behaviours and clinical outcomes in adults with diabetes or related metabolic disorders: A systematic review and meta-analysis protocol

Journal:	*BMJ Open*
Manuscript ID	bmjopen-2021-056293
Article Type:	Protocol
Date Submitted by the Author:	09-Aug-2021

Complete List of Authors:	Mueller, Julia; University of Cambridge, MRC Epidemiology Unit<br>Ahern, Amy; University of Cambridge, MRC Epidemiology Unit<br>Sharp, Stephen; University of Cambridge, MRC Epidemiology Unit<br>Richards, Rebecca; University of Cambridge, MRC Epidemiology Unit<br>Birch, Jack; University of Cambridge, MRC Epidemiology Unit<br>Davies, Alan; The University of Manchester, Division of Informatics, Imaging & Data Sciences<br>Griffin, Simon; University of Cambridge, MRC Epidemiology Unit; University of Cambridge, Department of Public Health and Primary Care

Keywords:	DIABETES & ENDOCRINOLOGY, HEALTH SERVICES ADMINISTRATION & MANAGEMENT, SOCIAL MEDICINE

SCHOLARONE™
Manuscripts

The association between patient activation, self-management behaviours and clinical outcomes in adults with diabetes or related metabolic disorders: A systematic review and meta-analysis protocol

*Julia Mueller**[1] julia.mueller@mrc-epid.cam.ac.uk

Amy L. Ahern[1] amy.ahern@mrc-epid.cam.ac.uk

Stephen J. Sharp[1] stephen.sharp@mrc-epid.cam.ac.uk

Rebecca Richards[1] rebecca.richards@mrc-epid.cam.ac.uk

Jack M. Birch[1] jack.birch@mrc-epid.cam.ac.uk

Alan Davies[2] alan.davies-2@manchester.ac.uk

Simon J. Griffin[1,3] profgp@medschl.cam.ac.uk

[1] MRC Epidemiology Unit, University of Cambridge, Cambridge, UK.

[2] Division of Informatics, Imaging & Data Sciences, University of Manchester, Manchester, Uk.

3 Department of Public Health and Primary Care, University of Cambridge, Cambridge, UK.

* Corresponding author: Julia Mueller, MRC Epidemiology Unit, University of Cambridge School of Clinical Medicine, Box 285 Institute of Metabolic Science, Cambridge Biomedical Campus, Cambridge, CB2 0QQ; Phone: +44 (0) 1223 769138

Keywords: Patient activation; diabetes; obesity; cardiovascular disease; self-management

Word count: 3823 words

For peer review only - http://bmjopen.bmj.com/site/about/guidelines.xhtml

Abstract

Introduction: Diabetes and related metabolic disorders such as obesity and cardiovascular diseases (CVD) are a growing global issue. Equipping individuals with the necessary 'knowledge, skills and confidence to self-manage their health' (i.e. patient activation [PAct]) may lead to improvements in health outcomes. Evidence on the relationship of PAct with self-management behaviours and clinical outcomes has not been synthesised systematically. Additionally, it is unclear whether existing evidence allows us to assume a causal relationship. We aim to synthesise and critically appraise evidence on the relationship between PAct and self-management behaviours and clinical outcomes of people living with diabetes and related metabolic disorders.

Methods and analysis: The protocol is based on guidance on Preferred Reporting Items for Systematic Review and Meta-analysis Protocols (PRISMA-P). We will search Medline, Embase, CENTRAL, PsycInfo, Web of Science, and CINAHL using search terms related to patient activation, diabetes, prediabetes, obesity, and cardiovascular disease. Any quantitative study design is eligible provided studies assess the association between PAct and clinical outcomes and/or self-management behaviours of diabetes and related metabolic disorders. Outcomes include behavioural (e.g. diet) and clinical (e.g. blood pressure) outcomes. Two reviewers will independently screen titles/abstracts and full texts and assess risk of bias. One reviewer will extract data, with independent checking by a second reviewer. We will critically assess the level of evidence available for assuming a causal association between PAct and outcomes. Data permitting, we will use the Hunter-Schmidt random-effects method to meta-analyse correlations across studies.

Ethics and dissemination: Ethical approval is not required. The review will be disseminated in the form of a peer-reviewed journal article, at conferences and other presentations (e.g. webinars). The findings of the review will be of interest to clinical commissioning groups, policy makers and intervention deliverers/developers.

Registration: Prospero registration number: CRD42021230727

Article Summary

Strengths and limitations of this study

- This review assesses whether patient activation is a proxy measure for wider health outcomes, and includes a broad range of clinical and behavioural outcomes

For peer review only

- It uses a comprehensive search strategy with a broad range of relevant databases, including databases that allow insight into grey literature (e.g. conference abstracts, theses)
- We will conduct a thorough critical appraisal of the evidence, based on a systematic procedure adapted from previous reviews, to assess whether evidence supports causal assumptions
- We expect high heterogeneity across studies, which may make meta-analysis infeasible or difficult to interpret

Background

Excess body weight is a major risk factor for chronic health problems such as diabetes mellitus and cardiovascular disease (CVD).[1,2] Diabetes and related metabolic disorders (e.g. obesity and CVD) are linked to poor patient outcomes such as reduced quality of life [e.g. 3] as well as increased direct and indirect economic costs, mainly due to medication, hospitalisations, disability and loss of productivity.[4–10] Equipping individuals with the necessary knowledge, skills and confidence to achieve sustained changes in their behaviour and self-manage their health and healthcare may lead to improvements in health-related outcomes and reduced hospitalisation and costs.[11–15]

The construct encompassing patients' knowledge, confidence and skills for self-management has been termed 'patient activation' (PAct).[16] A recent systematic review on PAct in adults with chronic conditions identified two measures of PAct, the Patient Activation Measure (PAM) and Patient Assessment of Chronic Illness Care (PACIC), which includes a sub-domain on PAct.[17] The PAM is the most commonly used instrument to assess PAct. It is a self-report measure with either 22 or 13 items (short form).[16,18] PAM scores range from 0 to 100 with higher scores indicating higher activation. PAM scores are categorised by four stages of activation: stage 1 (≤47.0) and stage 2 (47.1-55.1) are categorised as low activation levels, and stage 3 (55.2-67.0) and stage 4 (≥67.1) are categorised as high activation levels. The PAM is widely used in healthcare delivery and evaluation.[19,20] For example, within the UK National Health Service (NHS) the PAM is used for population segmentation and risk stratification in order to target and tailor interventions.[19] General Practitioner practices have used the PAM to tailor their diabetes review process such that participants with lower activation levels receive longer appointments than those with high activation levels.[20] PAM scores are also used to allocate different interventions to individuals with different activation levels. As such, it is important to understand how the PAM (and other PAct measures) are associated with clinical outcomes and self-management behaviours.

For peer review only - http://bmjopen.bmj.com/site/about/guidelines.xhtml

PAct and self-management behaviours relevant to diabetes and related metabolic disorders

There is some evidence to indicate that PAct is associated with self-reported self-management behaviours relevant to diabetes and related metabolic disorders, such as eating a healthy diet, being physically active, adhering to medication, and smoking cessation.[16,18,21–26] For some outcomes, such as self-reported physical activity, the relationship with PAct appears consistent.[16,18,21,22,24,25] For other outcomes the relationship is less clear. For example, some studies have found no significant association between PAct and smoking,[21–23] and in Hibbard & Tusler's study, correlations with diet-related variables (e.g. self-reported fruit and vegetable consumption) seemed to vary depending on the population and the specific behaviour measured.[22] Although several studies have assessed associations between PAct and self-management behaviours, this evidence has, to our knowledge, not been synthesised in a systematic review.

PAct and clinical outcomes of diabetes and related metabolic disorders

Self-reported behavioural measures are prone to error (which may be correlated with error in the measure of PAct) and bias. Furthermore, it is not clear how associations between PAct and health behaviours translate into clinical outcomes. As the PAM is used in the evaluation of healthcare systems and interventions,[19] it is important to understand not only if this measure (and any other PAct measures) predict self-management behaviours (such as adhering to a healthy diet), but also how PAct measures relate to clinical outcomes.

Several studies have found significant associations between PAct and clinical outcomes such as HbA_{1C}, blood glucose, triglycerides, cholesterol and blood pressure. [23,26–30] However, the evidence base is heterogeneous and complex, with some studies finding no significant associations,[26,28] significant associations opposite to those hypothesised,[26] or inconsistent patterns across PAct levels (i.e. unclear dose-response relationships).[27] The relationship between PAct and objective clinical outcomes is therefore unclear and warrants further investigation and synthesis.

PAct as a causal factor for health outcomes

The concept of PAct is often used to inform intervention development to support patient self-management and participation and engagement in health care.[19] The underlying assumption is that increases in PAct cause improvements in health outcomes. It is therefore important to

For peer review only - http://bmjopen.bmj.com/site/about/guidelines.xhtml

understand not only whether there is an association between PAct and outcomes of diabetes and related metabolic disorders, but also whether there is evidence for a causal pathway.

Two systematic reviews have assessed the impact of interventions targeting PAct on diabetes outcomes and found some evidence for effects on glycaemic control and self-management behaviours.[31,32] However, many of the included interventions are complex and include several components, and formal mediation analyses to assess whether interventions effects were mediated by increases in PAct were not carried out. It is therefore difficult to ascertain whether interventions effected change through PAct or other mechanisms.

Findings from individual studies suggest PAct interventions can significantly decrease weight and blood pressure and improve glycaemic control in people with overweight or obesity,[33] as well as reducing risk factors for cardiovascular disease, such as smoking and lack of exercise.[34] However, to our knowledge, no systematic review has assessed the effects of PAct interventions for adults with overweight, obesity or CVD.

To conclude, although several studies have explored the association between PAct and clinical outcomes as well as self-management behaviours relevant to diabetes and related metabolic disorders, they have not yet been synthesised in the form of a systematic review. Moreover, it is unclear whether current evidence is sufficient to assume a causal link between PAct and outcomes. A systematic review of the literature is required to assess the association between PAct and outcomes of diabetes and related metabolic disorders, and to critically appraise the strength of this evidence.

Aims

The aims of this review are:

i. To systematically review and synthesise evidence on the association between PAct and self-management behaviours relevant to diabetes and related metabolic disorders (e.g. diet, physical activity).
ii. To systematically review and synthesise evidence on the association between PAct and clinical outcomes of diabetes and related metabolic disorders (e.g. blood pressure, HbA_{1c}).
iii. To critically appraise whether the evidence is sufficient to assume a causal role of PAct in improving clinical outcomes and self-management behaviours.

Methods

The protocol is based on guidance on conducting systematic reviews provided by the Centre for Reviews and Dissemination,[35] Preferred Reporting Items for Systematic Reviews and Meta-

For peer review only - http://bmjopen.bmj.com/site/about/guidelines.xhtml

Analyses (PRISMA),[36] and Preferred Reporting Items for Systematic Review and Meta-analysis Protocols (PRISMA-P).[327]

We will adopt a 2-phase approach, whereby the first phase will involve a systematic scoping of the literature. This will involve establishing a list of all studies (cross-sectional, longitudinal, intervention) that examine the relationship between PAct and outcomes. Depending on the studies found in Phase 1, we will then consider whether we are able to narrow down our review questions, e.g. by population (e.g. only diabetes populations), or study design.

Inclusion/exclusion criteria

Studies will be eligible if they include a measure of PAct (e.g. PAM, PACIC) and assess the association between PAct and clinical outcomes and/or self-management behaviours relevant to diabetes and related metabolic disorders, or if they assess the effect on such outcomes of interventions that explicitly target patient activation.

Population

We will include studies with samples consisting of adults (≥ 18 years old) who have diabetes or a related metabolic disorder. We defined “diabetes and related metabolic disorders” to include prediabetes, diabetes (including but not limited to type 1/type 2 diabetes and gestational diabetes), obesity, and CVD. We define prediabetes as a state with glycaemic levels above ‘normal’ but below cut-offs for a diagnosis of diabetes. As such, we will include any studies that describe their population as being diagnosed with prediabetes, impaired glucose tolerance, glucose intolerance, impaired fasting glycaemia, borderline diabetes, non-diabetic hyperglycaemia, or similar.[38] We will not apply any specific criteria (e.g. cut-offs for impaired fasting glucose or impaired glucose tolerance). We define CVD as any conditions affecting the heart or blood vessels, including (but not limited to): coronary heart disease (angina, heart attacks, heart failure), strokes and transient ischaemic attacks, peripheral arterial disease, and aortic disease. Studies will be eligible if they include one or more of these disease types in a broader sample if results are reported separately for our population of interest.

Interventions

We will include studies of varying designs, including intervention studies (see ‘Study designs’). Where we include intervention studies, any type of intervention will be eligible as long as PAct is measured and the study reports on its association with our pre-defined outcomes, since the primary

For peer review only - http://bmjopen.bmj.com/site/about/guidelines.xhtml

For peer review only

aim of the review is not to assess the effectiveness of a particular type of intervention but to assess the relationship between PAct and outcomes.

If an intervention study reports intervention effects on PAct and effects on other specified outcomes but does not report on the association between PAct and outcomes, we will include the study only if (i) the intervention explicitly aims to increase PAct or is described as targeting patients' knowledge, confidence and skills for self-management (as opposed to interventions that target related but different constructs such as self-efficacy) and (ii) increasing PAct is a key, main component of the intervention (i.e. studies will be excluded if PAct components form part of a complex intervention with other components). Such studies will be excluded from quantitative synthesis, but will be included in narrative synthesis as they can provide evidence of an association between PAct and outcomes.

Comparators

Where we include intervention studies, any type of comparator will be eligible (as well as observational studies or other intervention studies with no comparator, e.g. pre-post studies).

Exposure

We will include only studies that include a measure of PAct (e.g. PAM, PACIC, or other measures of PAct). We will not include studies that measure related constructs (e.g. confidence, or self-efficacy) if the measures do not explicitly purport to assess patient activation.

Outcomes

We will focus on clinical outcomes and self-management behaviours that are shared between diabetes and related metabolic disorders. Both self-reported and objectively measured outcomes will be eligible. We will include studies that measure at least one of the following outcomes:

Clinical outcomes

- HbA_{1C} level / glycaemic control
- Systolic blood pressure / diastolic blood pressure
- Low-density lipoprotein (LDL) / High-density lipoprotein (HDL) / Total cholesterol
- Serum triglycerides
- Body Mass Index (BMI) / body weight

Self-management behaviours

- Outcomes related to diet (e.g. fruit/vegetable consumption, following a low-fat diet)

For peer review only - http://bmjopen.bmj.com/site/about/guidelines.xhtml

- Outcomes related to physical activity (e.g. step counts, following a regular exercise schedule, frequency of physical activity)
- Outcomes related to smoking (e.g. smoking status)
- Outcomes related to alcohol consumption (e.g. alcohol consumption, frequency or amounts)
- Medication adherence

Study design

We will include original primary research articles. We will include all study designs, including cross-sectional, longitudinal and intervention (e.g. randomised controlled trials (RCTs), pre-post comparison studies) as long as studies report on the association between PAct and one of the specified outcomes. We will exclude study protocols, literature reviews/meta-analyses, qualitative studies, and studies not reporting on empirical data.

Language and date

We will include studies in any language, subject to local translation resources. Searches will not be limited by date.

Publication status

We will endeavour to include both published and unpublished materials (e.g. abstracts, theses) to reduce the impact of publication bias.[35]

Information sources and search strategy

Databases

The following databases will be searched:

- Medline
- Embase
- CENTRAL
- PsycInfo
- Web of Science
- CINAHL

Search strategy

The search strategy (Table 1) was devised with the help of a medical librarian. The search strategy is outlined in Table 1, and an example of the proposed search strategy is shown in Appendix A. References of included studies will be hand-searched for further eligible studies. Searches will be re-run prior to the final analysis. To identify relevant grey literature, we will search the Health

For peer review only - http://bmjopen.bmj.com/site/about/guidelines.xhtml

Management Information Consortium (HMIC) database, ZETOC (using the conference search), and the British Library Integrated Catalogue.

Table 1 Search terms for the systematic review.

Concept	Free text	MeSH
Patient activation	“patient* activation*”<br>measure* ADJ5 “patient activation”<br>PAM?22*<br>PAM?13*<br>PAM??13*<br>PAM??22*<br>"Patient Assessment of Chronic Illness Care*"<br>PACIC*	
Diabetes	Diabet*<br>T2DM<br>T1DM<br>(non insulin* depend* or non insulin depend* or non insulin?depend* or non insulin?depend)<br>IDDM or NIDDM or MODY<br>T1D or T2D	exp Diabetes Mellitus, Type 2/ or exp Diabetes Mellitus/ or exp Diabetes Mellitus, Type 1<br><br>exp diabetes insipidus
Prediabetes	Pre?diabet*<br>Borderline ADJ3 diabet*<br>Impair* ADJ3 glucose<br>“Non-diabetic hyperglyc?emi*”<br>Glucose ADJ3 intoleran*	exp Prediabetic State/ or exp Glucose Intolerance/
Obesity/Overweight	Obes*<br>Overweight<br>“over weight”<br>Body ADJ3 weight<br>“body weight”<br>Adiposit*<br>Weight adj3 (gain* or loss* or chang* or control* or maintain* or reduc* or manag*)	exp Obesity/ OR exp Overweight/ OR exp Body Weight/ OR exp Adiposity/ or exp body mass index/

For peer review only - http://bmjopen.bmj.com/site/about/guidelines.xhtml

	Bmi or body mass ind*	
Heart disease	Heart* OR<br>cardiovascular<br>OR<br>coronary OR cardio* OR cardiac*	exp Heart Diseases/<br>OR exp<br>Cardiovascular<br>Diseases/<br>exp Coronary<br>Disease/ OR exp<br>heart failure/

Data management and selection process

Citations returned through the database search will be exported into Covidence and de-duplicated for screening. Two reviewers will independently screen titles and abstracts for eligibility, and will then read full texts of selected citations to further assess eligibility. Any disagreements will be resolved by a third independent reviewer. Interrater reliability will be assessed using Cohen's Kappa.[39]

Data extraction

Initially, we will extract study information into a table to summarise broad study characteristics. We will use this to assess the available evidence and decide whether to narrow down our review objectives (e.g. to a specific disease population). Data from included studies will be extracted into a data extraction sheet (draft shown in Appendix B). The data extraction sheet is adapted from the Cochrane data collection form for RCTs and non-RCTs[40] and was also informed by the STROBE checklist of items that should be included in reports of observational studies,[41] the CONSORT statement,[42] and the risk of bias tools we used (Table 2).

Data to be extracted include details regarding study design, population, sample size, details about the intervention if relevant, methods used to assess outcomes, and details on the reported association between PAct and outcomes (including effect size, whether adjusted or unadjusted, and what covariates were included in adjusted models). One reviewer will extract data and one reviewer will independently check this for accuracy and completeness. The data extraction sheet will be pilot-tested by at least 2 reviewers on three studies. Any issues will be discussed and the sheet will be updated accordingly.

For peer review only

Risk of bias / Quality appraisal

We will use two different tools to assess risk of bias, depending on study design (Table 2).

Table 2 Risk of bias tools to be used in the review, depending on study design.

Study design	Risk of bias tool
Randomised controlled trial*	RoB 2: A revised Cochrane risk-of-bias tool for randomized trials[43]
Observational studies	Risk of Bias Assessment Tool for Nonrandomized Studies (RoBANS)[44]

** RCTs that have been analysed as a cohort study (i.e. reporting on the association between PAct and outcomes, regardless of study group allocation), will be assessed using the RoBANS tool. If the data we extract depend on study group allocation, we will use the RoB 2 tool.*

Each study will be appraised by two independent review authors. Reviewers will discuss any discrepancies until they reach a consensus, consulting a third reviewer if required. Any potential sources of bias or methodological limitations not covered by the tools will be noted by the reviewers. Each study will be assigned an overall risk rating of high, low or unclear (RoBANS tool) or high/low/some concerns (ROB 2). Risk of bias assessments will be used to determine the level of evidence (see section on 'Levels of evidence'). For the purpose of determining the level of evidence, risk of bias will be dichotomised into high/low risk (for RoBANS, 'unclear' and 'high' and for ROB2, 'some concerns' and 'high' will be amalgamated).

Data synthesis and analysis

The study selection process will be depicted in a PRISMA diagram. Key results will be presented in form of a table summarising study characteristics. Risk of bias assessments will also be provided in a table.

Narrative synthesis: Levels of evidence

A key output of this review will be an assessment of the level of evidence available for assuming a causal association between PAct and self-management behaviours as well as clinical outcomes of diabetes and related metabolic disorders. The 'level of evidence' will be a composite measure, based on the strength of the study design/analysis, the quality of the study, sample size, and the consistency of the findings, adapted from an approach used in a previous systematic review.[45]

Table 3 shows the types of study designs, coupled with different types of analyses, that could provide evidence for a causal assumption, grouped into different categories based on their

For peer review only - http://bmjopen.bmj.com/site/about/guidelines.xhtml

suitability to support this assumption. If we encounter any unanticipated study designs/analyses, we will discuss this within the review team to assign the appropriate categorisation.

Once study designs and analyses have been categorised according to Table 3 and once studies have been assigned a risk of bias appraisal, we will use Figure 1 to assign a level of evidence, depending on the consistency of the findings across studies. Findings will be considered to be consistent if at least two thirds (66.6%) of the highest quality studies are reported to have significant results in the same direction.[45]

Table 3. Categorisation of the suitability of different study designs (coupled with different analyses) to draw conclusions regarding a causal association between PAct and outcomes of diabetes and related metabolic disorders. PAct = patient activation

**Possible study designs + analyses**	**Suitability of study design and analyses**	**Rationale**
RCTs with causal mediation analysis to assess whether PAct mediates intervention effects	strong	RCTs are the only study design that allow causal mediation analysis to identify the mechanisms by which interventions exert their effects[46]
Cohort studies / RCTs or other intervention studies that assess the association between PAct and subsequent outcomes	moderate	RCTs and longitudinal observational studies can provide temporal insights into the association between PAct and outcomes, which gives some indication of causality.[47] If an RCT examines the association between PAct and outcomes independent of study group allocation, randomisation has no bearing; analyses & findings are therefore akin to cohort studies.
RCTs that do not report on the association between PAct and outcomes but that show intervention effects on outcomes AND intervention effects on PAct, AND the intervention explicitly, mainly addresses PAct	moderate	RCTs provide insight into causal effects of interventions on outcomes. If an intervention explicitly addresses PAct and there is evidence that the intervention influenced both PAct and outcomes, this provides indication for a causal mechanism of PAct on outcomes (though not definitive).
Observational cross-sectional studies	weak	In cross-sectional designs, the time order of effects cannot be determined and therefore causality cannot be inferred.[48]
Intervention studies that are not RCTs (e.g. pre-post studies) and that do not report on the association between PAct and outcomes but that show changes in outcomes AND changes in PAct.	weak	Pre-post designs have the strength of temporality to indicate outcomes might be impacted by an intervention, but due to lack of randomisation causality cannot be inferred.[49]

For peer review only - http://bmjopen.bmj.com/site/about/guidelines.xhtml

For peer review only

For peer review only - http://bmjopen.bmj.com/site/about/guidelines.xhtml

[Insert Figure 1 here]

Figure 1. Levels of evidence. To be used in conjunction with Table 3. Note: studies including ≤ 250 participants or studies not providing sample size justifying a smaller sample size are considered 'small', studies including >250 participants are considered 'large'. Findings are considered consistent if at least two thirds (66.6%) of the highest quality studies are reported to have significant results in the same direction.

Narrative synthesis: Harvest Plot

If meta-analysis is not feasible and we cannot produce forest plots, we will create Harvest Plots to synthesise and depict our findings, adapted from the approach used by Ogilvie et al.[50] The plot will consist of a matrix with one row per outcome, and one column (for the assumption that there is a causal relationship between PAct and outcomes). Each study will be represented by a bar in each row for which that study reported relevant evidence. The strength of the study design and the analysis will be represented by the height of the bar, with higher bars indicating more suitable design and analysis. Studies using self-reported outcomes will be represented by a grey bar, while bars for studies using objective measures will be black. Each bar will be annotated with the quality appraisal for that study (e.g. high, low or unclear) and the sample size.

Meta-analysis

Meta-analysis will be undertaken if studies are considered sufficiently similar in their research questions, designs and outcomes. From each study, we will extract effect sizes for the association between PAct and the pre-specified outcomes. We will extract unadjusted and adjusted associations, and synthesise these separately. Regression coefficients from models with different sets of covariates represent different parameters and cannot be combined meaningfully.[51] We will therefore initially assess which covariates are included in adjusted models and, if there is agreement between models in terms of key covariates, we will synthesise coefficients across models (even if model specifications are not completely identical). If there is insufficient agreement between models in terms of covariates, we will include adjusted associations in the narrative synthesis, and focus on unadjusted associations in the quantitative synthesis.

We expect studies to report a wide range of different estimates of the association between PAct and outcomes. We will therefore initially convert different measures of the association to the Pearson Product Moment Correlation using the formulae in Table 4, because the correlation coefficient is an easily interpretable effect size to assess the strength of association between two variables. Some studies may report only odds ratios (as PAct scores are often dichotomised into high/low and clinical outcomes are often dichotomised into within/not within normal range). If studies report odds ratios,

For peer review only - http://bmjopen.bmj.com/site/about/guidelines.xhtml

we will construct contingency tables based on information about percentages of PAct levels and outcomes and use these tables to calculate χ^2 values, which can then be transformed to r.

We will use a random-effects approach, because we assume that the population effect sizes vary randomly from study to study (rather than assuming the population effect size is the same for all studies), e.g. due to differences in age, socioeconomic status, geographic location, or disease. Random effects meta-analysis allows inferences beyond the studies included in the analysis.[52] We will use the Hunter-Schmidt random-effects method to synthesise correlations across studies, because this method produces more accurate estimates than the Hedges-Olkin and Rosenthal-Rubin methods (except when the average population effect size is very large).[52] Effect sizes from cross-sectional and longitudinal studies will be synthesised separately.

If a study reports more than one estimate of association for a particular combination of exposure and outcome, we will select the estimated association based on the longest duration of follow-up or the most precise measure of the outcome. If it is not possible to discern this, within-study meta-analytic calculations will be used to obtain a single effect size, to maintain the statistical assumption of independence necessary for a meta-analysis. If the effect sizes are based on different sample sizes, the average sample size will be calculated and used for subsequent analyses. **Error! Reference source not found.**

Table 4. Formulae to convert different measures of effect to Pearson's r, based on Wolf (1986),[53] *Friedman (1982),*[54] *and Hoeve at al. (2009)*[55]

Statistic to be converted	Formula for transforming to Pearson Product Moment Correlation r	Notes
T	$\sqrt{\frac{t^2}{t^2+df}}$	
F(df=1)	$\sqrt{\frac{F}{F+df_D}}$	Use only for comparing two group means (df=1)<br>df_D: df of the denominator
F(df>1)	$\sqrt{\frac{df_N(F-1)}{df_N+df_D}}$	df_N: df of the numerator (k-1)<br>df_D: df of the denominator (N-k)
χ^2 (df=1)	$\sqrt{\frac{\chi^2}{n}}$	Use only for 2x2 frequency tables (df=1)
χ^2 (df>1)	$\sqrt{\frac{\chi^2}{\chi^2+N}}$	
D	$\sqrt{\frac{d}{d^2+4}}$	
Φ	(1) $\chi^2 = \phi^2 * N$<br>(2) Use equation for χ^2(df=1) or χ^2 (df>1)	

For peer review only - http://bmjopen.bmj.com/site/about/guidelines.xhtml

Exploration of heterogeneity

If sufficient studies are available, we will perform meta-regression to assess whether the effect size varies with study characteristics, including:

- Studies with different populations (diabetes/prediabetes, obesity, CVD)
- Self-reported vs. objectively measured outcomes
- Clinical vs. behavioural outcomes

Meta-regression will be performed on correlations transformed according to the Fisher z-transformation.[56]

Sensitivity analyses

Sensitivity analysis will be performed excluding studies that are categorised as high risk of bias, to assess whether findings are unduly influenced by these studies.

Assessment of heterogeneity and reporting bias

To assess heterogeneity, we will report the I^2 statistic with a 95% confidence interval, as well as outcomes from the test for heterogeneity (Q-statistic and associated p-value). For I^2, we will categorise heterogeneity as low (0%–30%), moderate (30%–60%), substantial (60%–90%) and considerable (90%–100%).[57] To assess publication bias, we will construct funnel plots, plotting the mean correlation against study sample sizes as well as the residual standard deviation of r against the sample size.

Patient and Public Involvement

We shared a lay summary of the review protocol with an established patient and public involvement (PPI) panel. Feedback was positive, with panel members commenting that they think the review will be useful, particularly within NHS services. Panel members also made recommendations for our dissemination strategy to help us reach a wider audience. After completing the review, we will seek feedback from the PPI panel on a lay summary of the review findings and on our dissemination plan. The protocol was further reviewed by a GP partner from NHS Cambridgeshire and Peterborough CCG, who has particular expertise in person centred, collaborative care and long-term conditions.

Ethics and dissemination

Ethical approval is not required for this systematic review. The review will be disseminated in the form of a peer-reviewed journal article, at conferences and other presentations (e.g. webinars), as

For peer review only - http://bmjopen.bmj.com/site/about/guidelines.xhtml

For peer review only

well as more publicly accessible formats such as blog posts, social media posts, and, if suitable, a press release. The findings of the review will be of interest to clinical commissioning groups, policy makers and intervention deliverers/developers that currently use, or plan to use, the PAM or other measures of PAct to tailor and allocate interventions for diabetes and related metabolic disorders. It will also be of relevance to those using measures of PAct to evaluate intervention effectiveness and healthcare performance, as it will provide an indication of how well PAct predicts outcomes for diabetes and related metabolic disorders.

Amendments

Amendments made will be noted in a pre-specified section of the protocol (rather than being incorporated into the protocol), with the date and rationale. Amendments will also be uploaded to Prospero. Since commencing title/abstract screening, we have made one amendment (Table 5).

Table 5. Amendments to the protocol.

Date	Change	Rationale
29/01/2021	Removed “Life expectancy/ total survival” from the list of outcomes	After discussion within the team, we decided this outcome does not align well with the other included outcomes. The other outcomes give an indication of how well people self-manage their condition, whereas life expectancy/survival is a wider measure that gives less insight into self-management specifically. Moreover, there are unlikely to be many studies with sufficiently long follow-up to provide any meaningful assessment of survival in this context, and even if there was a study with very long follow-up, we would then be relying on an assumption that the patient activation exposures measured at baseline do not change over time.

Author contributions

JM drafted the manuscript, with regular input from all co-authors. All authors read, provided feedback and approved the manuscript prior to submission. JM, AA, SG, RR, JB and AD contributed to the development of the selection criteria, the risk of bias assessment strategy and data extraction criteria. SS provided statistical expertise.

Funding statement

This research received no specific grant from any funding agency in the public, commercial or not-for-profit sectors.

For peer review only - http://bmjopen.bmj.com/site/about/guidelines.xhtml

Competing interests statement

None declared.

Acknowledgements

We would like to thank Dr Isla Kuhn for reviewing our protocol and helping us refine our search strategy. We also thank our PPI panel and Dr Mark Brookes for reviewing our protocol and providing feedback and comments.

References

1 Van Gaal LF, Mertens IL, De Block CE. Mechanisms linking obesity with cardiovascular disease. Nature. 2006;**444**:875–80. doi:10.1038/nature05487

2 Al-Goblan AS, Al-Alfi MA, Khan MZ. Mechanism linking diabetes mellitus and obesity. *Diabetes, Metab Syndr Obes Targets Ther* 2014;**7**:587–91. doi:10.2147/DMSO.S67400

3 Speight J, Holmes-Truscott E, Hendrieckx C, *et al.* Assessing the impact of diabetes on quality of life: what have the past 25 years taught us? *Diabet Med* 2020;**37**:dme.14196. doi:10.1111/dme.14196

4 Mata-Cases M, Casajuana M, Franch-Nadal J, *et al.* Direct medical costs attributable to type 2 diabetes mellitus: a population-based study in Catalonia, Spain. *Eur J Heal Econ* 2016;**17**:1001–10. doi:10.1007/s10198-015-0742-5

5 Giorda CB, Rossi MC, Ozzello O, *et al.* Healthcare resource use, direct and indirect costs of hypoglycemia in type 1 and type 2 diabetes, and nationwide projections. Results of the HYPOS-1 study. *Nutr Metab Cardiovasc Dis* 2017;**27**:209–16. doi:10.1016/j.numecd.2016.10.005

6 Bain SC, Bekker Hansen B, Hunt B, *et al.* Evaluating the burden of poor glycemic control associated with therapeutic inertia in patients with type 2 diabetes in the UK. *J Med Econ* 2020;**23**:98–105. doi:10.1080/13696998.2019.1645018

7 Yazdanyar A, Newman AB. The Burden of Cardiovascular Disease in the Elderly: Morbidity, Mortality, and Costs. Clin. Geriatr. Med. 2009;**25**:563–77. doi:10.1016/j.cger.2009.07.007

8 Tarride JE, Lim M, DesMeules M, *et al.* A review of the cost of cardiovascular disease. *Can J*

For peer review only - http://bmjopen.bmj.com/site/about/guidelines.xhtml

For peer review only

Cardiol 2009;**25**:e195–202. doi:10.1016/S0828-282X(09)70098-4

9 Einarson TR, Acs A, Ludwig C, *et al.* Economic Burden of Cardiovascular Disease in Type 2 Diabetes: A Systematic Review. Value Heal. 2018;**21**:881–90. doi:10.1016/j.jval.2017.12.019

10 Tremmel M, Gerdtham UG, Nilsson PM, *et al.* Economic burden of obesity: A systematic literature review. Int. J. Environ. Res. Public Health. 2017;**14**. doi:10.3390/ijerph14040435

11 Tay JHT, Jiang Y, Hong J, *et al.* Effectiveness of lay-led, group-based self-management interventions to improve glycated hemoglobin (HbA1c), self-efficacy, and emergency visit rates among adults with type 2 diabetes: A systematic review and meta-analysis. *Int J Nurs Stud* 2020;:103779. doi:10.1016/j.ijnurstu.2020.103779

12 Zhao Q, Chen C, Zhang J, *et al.* Effects of self-management interventions on heart failure: Systematic review and meta-analysis of randomized controlled trials. Int. J. Nurs. Stud. 2020;**110**:103689. doi:10.1016/j.ijnurstu.2020.103689

13 Newman S, Steed L, Mulligan K. Self-management interventions for chronic illness. In: *Lancet*. Elsevier 2004. 1523–37. doi:10.1016/S0140-6736(04)17277-2

14 Galani C, Schneider H. Prevention and treatment of obesity with lifestyle interventions: Review and meta-analysis. Int. J. Public Health. 2007;**52**:348–59. doi:10.1007/s00038-007-7015-8

15 Zhang D, Cogswell ME, Wang G, *et al.* Evidence of Dietary Improvement and Preventable Costs of Cardiovascular Disease. Am. J. Cardiol. 2017;**120**:1681–8. doi:10.1016/j.amjcard.2017.07.068

16 Hibbard JH, Stockard J, Mahoney ER, *et al.* Development of the Patient Activation Measure (PAM): Conceptualizing and Measuring Activation in Patients and Consumers. *Health Serv Res* 2004;**39**:1005–26. doi:10.1111/j.1475-6773.2004.00269.x

17 Newland P, Lorenz R, Oliver BJ. Patient activation in adults with chronic conditions: A systematic review. *J Health Psychol* Published Online First: 2020. doi:10.1177/1359105320947790

18 Hibbard JH, Mahoney ER, Stockard J, *et al.* Development and testing of a short form of the patient activation measure. *Health Serv Res* 2005;**40**:1918–30. doi:10.1111/j.1475-6773.2005.00438.x

19 Hibbard J, Gilburt H. Supporting people to manage their health: An introduction to patient

For peer review only

For peer review only - http://bmjopen.bmj.com/site/about/guidelines.xhtml

activation. 2014.
https://www.kingsfund.org.uk/sites/default/files/field/field_publication_file/supporting-people-manage-health-patient-activation-may14.pdf

20 NHS England. Patient activation and PAM FAQs. https://www.england.nhs.uk/personalisedcare/supported-self-management/patient-activation/pa-faqs/ (accessed 9 Sep 2020).

21 Rask KJ, Ziemer DC, Kohler SA, *et al.* Patient activation is associated with healthy behaviors and ease in managing diabetes in an indigent population. *Diabetes Educ* 2009;**35**:622–30. doi:10.1177/0145721709335004

22 Hibbard JH, Tusler M. Assessing activation stage and employing a 'next steps' approach to supporting patient self-management. *J Ambul Care Manage* 2007;**30**:2–8. doi:10.1097/00004479-200701000-00002

23 Hendriks M, Rademakers J. Relationships between patient activation, disease-specific knowledge and health outcomes among people with diabetes; a survey study. *BMC Health Serv Res* 2014;**14**:393. doi:10.1186/1472-6963-14-393

24 Harvey L, Fowles JB, Xi M, *et al.* When activation changes, what else changes? The relationship between change in patient activation measure (PAM) and employees' health status and health behaviors. *Patient Educ Couns* 2012;**88**:338–43. doi:10.1016/j.pec.2012.02.005

25 Hibbard JH, Mahoney ER, Stock R, *et al.* Do Increases in Patient Activation Result in Improved Self-Management Behaviors? *Health Serv Res* 2007;**42**:1443–63. doi:10.1111/j.1475-6773.2006.00669.x

26 Greene J, Hibbard JH. Why does patient activation matter? An examination of the relationships between patient activation and health-related outcomes. *J Gen Intern Med* 2012;**27**:520–6. doi:10.1007/s11606-011-1931-2

27 Sacks RM, Greene J, Hibbard J, *et al.* Does patient activation predict the course of type 2 diabetes? A longitudinal study. *Patient Educ Couns* 2017;**100**:1268–75. doi:10.1016/j.pec.2017.01.014

28 Woodard LCD, Landrum CR, Amspoker AB, *et al.* Interaction between functional health literacy, patient activation, and glycemic control. Patient Prefer. Adherence. 2014;**8**:1019–24. doi:10.2147/PPA.S63954

For peer review only - http://bmjopen.bmj.com/site/about/guidelines.xhtml

29 Rogvi S, Tapager I, Almdal TP, *et al.* Patient factors and glycaemic control - associations and explanatory power. *Diabet Med* 2012;**29**. doi:10.1111/j.1464-5491.2012.03703.x

30 Remmers C, Hibbard J, Mosen DM, *et al.* Is patient activation associated with future health outcomes and healthcare utilization among patients with diabetes? *J Ambul Care Manage* 2009;**32**:320–7. doi:10.1097/JAC.0b013e3181ba6e77

31 Bolen SD, Chandar A, Falck-Ytter C, *et al.* Effectiveness and safety of patient activation interventions for adults with type 2 diabetes: Systematic review, meta-analysis, and meta-regression. J. Gen. Intern. Med. 2014;**29**:1166–76. doi:10.1007/s11606-014-2855-4

32 Almutairi N, Hosseinzadeh H, Gopaldasani V. The effectiveness of patient activation intervention on type 2 diabetes mellitus glycemic control and self-management behaviors: A systematic review of RCTs. Prim. Care Diabetes. 2020;**14**:12–20. doi:10.1016/j.pcd.2019.08.009

33 Barnason S, Zimmerman L, Schulz P, *et al.* Weight management telehealth intervention for overweight and obese rural cardiac rehabilitation participants: A randomised trial. *J Clin Nurs* 2019;**28**:1808–18. doi:10.1111/jocn.14784

34 Tinsel I, Siegel A, Schmoor C, *et al.* Encouraging Self-Management in Cardiovascular Disease Prevention. *Dtsch Arztebl Int* 2018;**115**:469–76. doi:10.3238/arztebl.2018.0469

35 Centre for Reviews and Dissemination. Systematic Reviews: CRD's guidance for undertaking reviews in health care. 2009.

36 Liberati A, Altman DG, Tetzlaff J, *et al.* The PRISMA statement for reporting systematic reviews and meta-analyses of studies that evaluate healthcare interventions: explanation and elaboration. *BMJ* 2009;**339**:b2700. doi:10.1136/bmj.b2700

37 Moher D, Shamseer L, Clarke M, *et al.* Preferred reporting items for systematic review and meta-analysis protocols (PRISMA-P) 2015 statement. *Syst Rev 2015 41* 2015;**4**:1–9. doi:10.1186/2046-4053-4-1

38 Diabetes UK. Prediabetes. https://www.diabetes.org.uk/preventing-type-2-diabetes/prediabetes (accessed 6 Jan 2021).

39 Cohen J. A Coefficient of Agreement for Nominal Scales. *Educ Psychol Meas* 1960;**20**:37–46. doi:10.1177/001316446002000104

40 The Cochrane Collaboration. Data extraction forms. 2020.https://dplp.cochrane.org/data-

For peer review only - http://bmjopen.bmj.com/site/about/guidelines.xhtml

extraction-forms

41 STROBE Statement. STROBE checklists. https://www.strobe-statement.org/index.php?id=available-checklists (accessed 16 Oct 2020).

42 Schulz KF, Altman DG, Moher D. CONSORT 2010 Statement: Updated guidelines for reporting parallel group randomised trials. *BMJ* 2010;**340**:698–702. doi:10.1136/bmj.c332

43 Sterne JAC, Savović J, Page MJ, *et al.* RoB 2: A revised tool for assessing risk of bias in randomised trials. *BMJ* 2019;**366**. doi:10.1136/bmj.l4898

44 Kim SY, Park JE, Lee YJ, *et al.* Testing a tool for assessing the risk of bias for nonrandomized studies showed moderate reliability and promising validity. *J Clin Epidemiol* 2013;**66**:408–14. doi:10.1016/j.jclinepi.2012.09.016

45 Van Sluijs EMF, McMinn AM, Griffin SJ. Effectiveness of interventions to promote physical activity in children and adolescents: Systematic review of controlled trials. *Br J Sports Med* 2008;**42**:653–7. doi:10.1136/bmj.39320.843947.BE

46 Lee H, Herbert RD, Lamb SE, *et al.* Investigating causal mechanisms in randomised controlled trials. *Trials* 2019;**20**:524. doi:10.1186/s13063-019-3593-z

47 Barnett ML, Hyman JJ. Challenges in interpreting study results The conflict between appearance and reality. 2006. doi:10.14219/jada.archive.2006.0405

48 Porta M. *Dictionary of Epidemiology*. Oxford: : Oxford University Press 2008.

49 Thiese MS. Observational and interventional study design types; an overview. *Biochem Medica* 2014;**24**:199–210. doi:10.11613/BM.2014.022

50 Ogilvie D, Fayter D, Petticrew M, *et al.* The harvest plot: A method for synthesising evidence about the differential effects of interventions. *BMC Med Res Methodol* 2008;**8**:8. doi:10.1186/1471-2288-8-8

51 Aloe AM. Inaccuracy of regression results in replacing bivariate correlations. *Res Synth Methods* 2015;**6**:21–7. doi:10.1002/jrsm.1126

52 Field AP. Meta-analysis of correlation coefficients: A Monte Carlo comparison of fixed- and random-effects methods. *Psychol Methods* 2001;**6**:161–80. doi:10.1037/1082-989X.6.2.161

53 Wolf F. *Meta-Analysis*. 2455 Teller Road, Newbury Park California 91320 United States of America : : SAGE Publications, Inc. 1986. doi:10.4135/9781412984980

For peer review only - http://bmjopen.bmj.com/site/about/guidelines.xhtml

54 Friedman H. Simplified Determinations of Statistical Power, Magnitude of Effect and Research Sample Sizes. *Educ Psychol Meas* 1982;**42**:521–6. doi:10.1177/001316448204200214

55 Hoeve M, Dubas JS, Eichelsheim VI, *et al.* The relationship between parenting and delinquency: A meta-analysis. J. Abnorm. Child Psychol. 2009;**37**:749–75. doi:10.1007/s10802-009-9310-8

56 Dingman HF, Perry NC. A Comparison of the Accuracy of the Formula for the Standard Error of Pearson "r" with the accuracy of Fisher's z-Transformation. *J Exp Educ* 1956;**24**:319–21. doi:10.1080/00220973.1956.11010555

57 Ryan R, Cochrane Consumers and Communication Review Group. Heterogeneity and subgroup analyses in Cochrane consumers and communication group reviews: planning the analysis at protocol stage. 2016.http://cccrg.cochrane.org (accessed 8 Jun 2020).

For peer review only

For peer review only - http://bmjopen.bmj.com/site/about/guidelines.xhtml

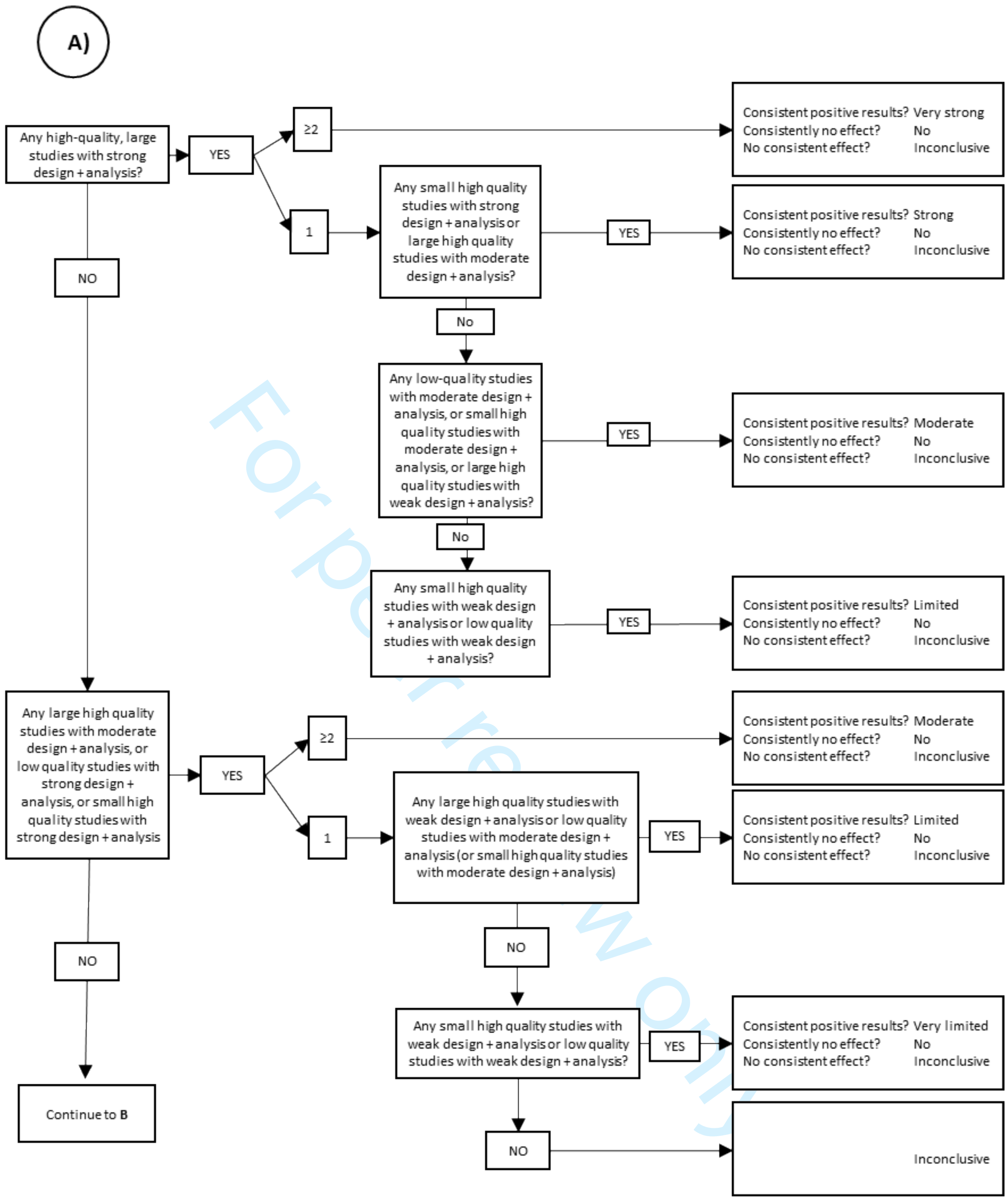

NOTE: studies including ≤ 250 participants or not providing sample size justifying a smaller sample size are considered 'small', studies including >250 participants are considered 'large.

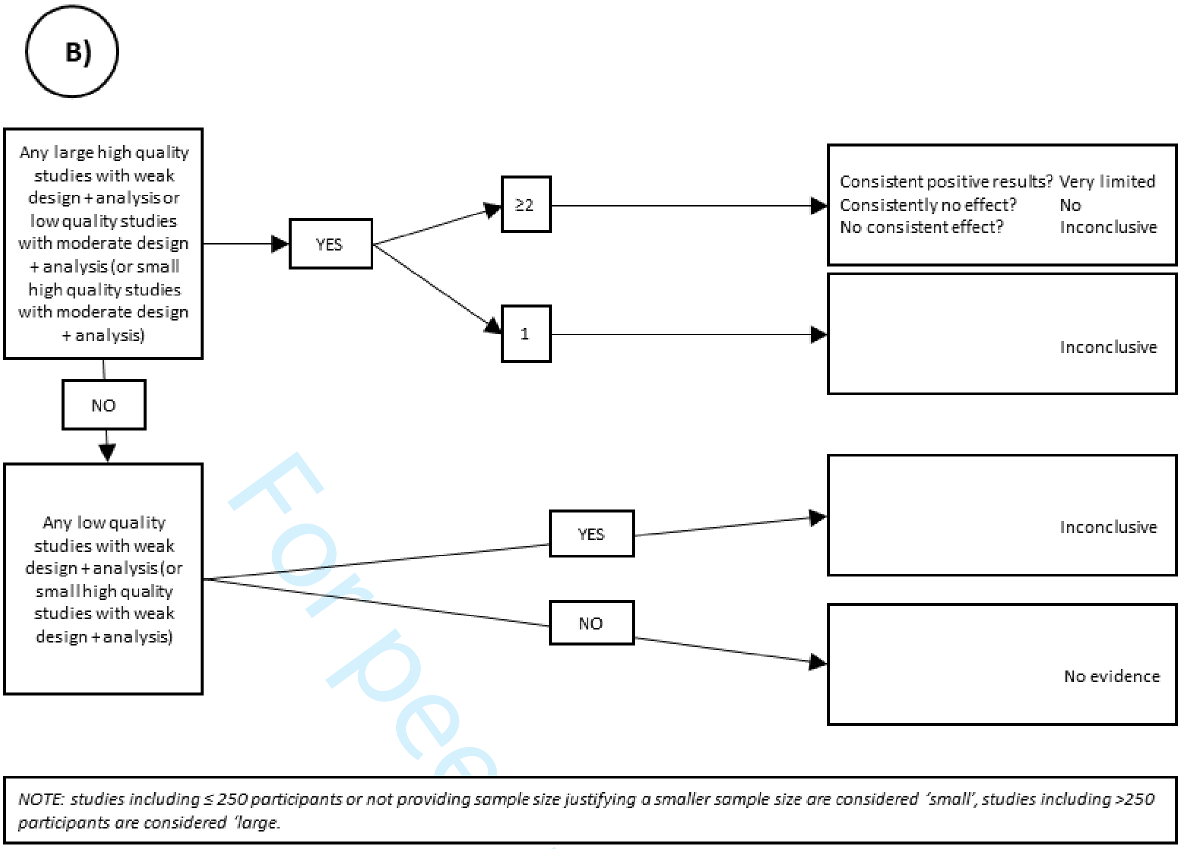

Figure 1. Levels of evidence. To be used in conjunction with ***Error! Reference source not found.****. Note: studies including ≤ 250 participants or studies not providing sample size justifying a smaller sample size are considered 'small', studies including >250 participants are considered 'large'. Findings are considered consistent if at least two thirds (66.6%) of the highest quality studies are reported to have significant results in the same direction.*

For peer review only

Appendix A: Example search strategy

Medline (Ovid)

1	("patient* activation*" or (measure* adj5 "patient activation") or PAM?22* or PAM?13* or PAM??13* or PAM??22* or "Patient Assessment of Chronic Illness Care*" or PACIC*).mp. [mp=title, abstract, original title, name of substance word, subject heading word, floating sub-heading word, keyword heading word, organism supplementary concept word, protocol supplementary concept word, rare disease supplementary concept word, unique identifier, synonyms]
2	(Diabet* or T2DM or T1DM or (non insulin* depend* or non insulin depend* or non insulin?depend* or non insulin?depend) or IDDM or NIDDM or MODY or T1D or T2D).mp. or exp Diabetes Mellitus, Type 2/ or exp Diabetes Mellitus/ or exp Diabetes Mellitus, Type 1/ or exp diabetes insipidus/ or exp Diabetes, Gestational/ [mp=title, abstract, original title, name of substance word, subject heading word, floating sub-heading word, keyword heading word, organism supplementary concept word, protocol supplementary concept word, rare disease supplementary concept word, unique identifier, synonyms]
3	(Pre?diabet* or (Borderline adj3 diabet*) or (Impair* adj3 glucose) or (Non-diabetic adj3 hyperglyc?emi*) or (Glucose adj3 intoleran*)).mp. or exp Prediabetic State/ or exp Glucose Intolerance/ [mp=title, abstract, original title, name of substance word, subject heading word, floating sub-heading word, keyword heading word, organism supplementary concept word, protocol supplementary concept word, rare disease supplementary concept word, unique identifier, synonyms]
4	(Obes* or Overweight or "over weight" or (Body adj3 weight) or "body weight" or Adiposit* or (Weight adj3 (gain* or loss* or chang* or control* or maintain* or reduc* or manag*)) or Bmi or body mass ind*).mp. or exp Obesity/ or exp Overweight/ or exp Body Weight/ or exp Adiposity/ or exp body mass index/ [mp=title, abstract, original title, name of substance word, subject heading word, floating sub-heading word, keyword heading word, organism supplementary concept word, protocol supplementary concept word, rare disease supplementary concept word, unique identifier, synonyms]
5	(Heart* or cardiovascular or coronary or cardio* or cardiac*).mp. or exp Heart Diseases/ or exp Cardiovascular Diseases/ or exp Coronary Disease/ or exp Heart Failure/ [mp=title, abstract, original title, name of substance word, subject heading word, floating sub-heading word, keyword heading word, organism supplementary concept word, protocol supplementary concept word, rare disease supplementary concept word, unique identifier, synonyms]
6	2 or 3 or 4 or 5
7	1 and 6

For peer review only - http://bmjopen.bmj.com/site/about/guidelines.xhtml

Appendix B: Data extraction sheet

Section 1: General meta-data

**Review title**	The association between patient activation, self-management behaviours and clinical outcomes in diabetes and related metabolic disorders: A systematic review					
**Study ID** *(surname of first author and year first full report of study was published e.g. Smith 2001)*						
**Date form completed** *(dd/mm/yyyy)*						
**Initials of person extracting data:**						
**Title:**						
**Author(s):**						
**Source:**						
**Date:**		**Vol:**		**Issue:**		**Pages:**
**Publication type** (e.g. full report, abstract)						

Section 2: Study eligibility

Study characteristics	Eligibility criteria	Eligibility criteria met?		
		Yes	No	Un-clear
Population	Adults ⍰18 years old) with diabetes or a related metabolic disorder (prediabetes, type 1 and type 2 diabetes , obesity, or CVD)	☐	☐	☐
Exposure	Includes a measure of patient activation (PAct)	☐	☐	☐
Outcomes	Includes at least one of the predefined outcomes, either clinical outcomes (HbA1C level/ glycaemic control, systolic blood pressure, diastolic blood pressure, low-density lipoprotein	☐	☐	☐

For peer review only

For peer review only - http://bmjopen.bmj.com/site/about/guidelines.xhtml

	(LDL), high-density lipoprotein (HDL), total cholesterol, serum triglycerides, BMI / weight, life expectancy/survival) or self-management behaviours (diet, physical activity, smoking, alcohol, medication adherence)			
Type of study	Original, primary research articles	☐	☐	☐
	Assesses the relationship between PAct and at least one of the defined outcomes	☐	☐	☐
	If no to the above: Is it an intervention study that reports intervention effects on PAct and effects on other specified outcomes AND (i) the intervention explicitly aims to increase patient activation or is described as targeting patients' knowledge, confidence and skills for self-management AND (ii) increasing patient activation is the main component of the intervention	☐	☐	☐
INCLUDE ☐		EXCLUDE ☐		
Reason for exclusion:				

DO NOT PROCEED IF STUDY EXCLUDED FROM REVIEW

Section 3: Objectives and design

**Objective:**	
**Setting:**	
**Country of origin:**	
**Start and end date:**	
**Study design**	
**Study population:**	
**Recruitment methods:**	
**Inclusion and exclusion criteria for participants:**	

For peer review only - http://bmjopen.bmj.com/site/about/guidelines.xhtml

Sample size:	
**Is a justification for the sample size provided (power calculation)?**	Yes/No (delete as appropriate)<br><br>Details:
**Withdrawals and exclusions:**	
**Attrition (i.e. loss to follow-up):**<br><br>**(For intervention studies, report per study group)**	

Section 2: Intervention details

Only complete Section 2 if it is an intervention study and we are interested in findings that depend on study group allocation. If it is an observational study, or an intervention study but the relevant data to extract pertain to the association between PAct and outcomes independent of study group allocation, skip to section 3.

	**Descriptions as stated in the report/paper**
**Randomisation and blinding:**	
**Sample size per group**	Intervention:<br>Control:
**Any indication for baseline differences between study groups?**	Yes/No/Unclear<br>Details:
**Comparison group description**	
**Intervention aim**	
**Is the explicit main aim of the intervention to increase patient activation or to target patients' knowledge, confidence and skills for self-management?**	*Yes/No/Unclear*<br>*(Delete as appropriate. Select No if the patient activation component forms part of a larger complex intervention).*
**Is patient activation the main component of the intervention?**	

For peer review only - http://bmjopen.bmj.com/site/about/guidelines.xhtml

**Intervention description**	
**Group or individual delivery**	
**Mode of delivery (e.g. web, face-to-face)**	
**Duration of intervention**	
**Timing (e.g. frequency, duration of each session)**	
**Providers (e.g. profession and training received)**	
**Intention to treat analysis?**	*Yes/No/Unclear (Delete as appropriate).*
**Any further notes:**	

For peer review only

Section 3: Outcomes & Measures

**PAct measure**	
**PAct measure used as continuous measure, ordinal (levels 1-4), or dichotomous (high/low)?**	Continuous/ordinal/dichotomous (delete as appropriate)
**Time points measured/reported (for all outcomes):**	
**Covariates:** *(Note: Only extract covariates that were included in models that assessed the association between PAct and the outcomes of interest as per review protocol)*	

Clinical outcomes

Note: If outcomes not measured, please insert "n/a"

	**How measured/defined (+unit of measurement)**	**Source (e.g. self-report, health records)**
HbA1C level/glycaemic control		
Systolic blood pressure, diastolic blood pressure		
Low-density lipoprotein (LDL)<br>High-density lipoprotein (HDL)<br>Total cholesterol		

For peer review only - http://bmjopen.bmj.com/site/about/guidelines.xhtml

Serum triglycerides		
BMI		
weight		
Life expectancy/survival		

Self-management behaviours

Note: If outcomes not measured, please insert "n/a"

	**Self-report? (Yes/No/Unclear)**	**How defined/measured?** ***e.g. "consuming 5 servings of fruit/veg per day (Yes/No)"***
Diet		
Physical activity		
Smoking		
Alcohol consumption		
Medication adherence		

For peer review only

Section 4: Analyses + Results

Please extract data for adjusted and unadjusted associations (i.e. associations just between PAct and the relevant outcome [=unadjusted], and those where a model such as a linear regression is used to control for confounders [=adjusted]). If extracting data for both adjusted and unadjusted associations, please add additional rows to the table (e.g. an additional row labelled 'Cross-sectional association with PAct' so that you have one for the adjusted and one for the unadjusted data).

If several time points are reported, extract data for the longest follow-up time point.

If several variables were used for the same outcome please copy and paste the table and add details for the respective variable (for example, create a second table for "diet", and add the variable.

If the format of the tables is unsuitable for the reported results, please paste the relevant results into the 'other/comments' section.

How were missing data handled? *(e.g. multiple imputation)*	

**Outcome: HbA1c/glycaemic control**						
**How measured/defined:**						
	**Statistical test**	**Adjusted/ unadjusted?**	**Covariates (if adjusted)**	**Effect size for the association** (e.g. [illegible] F, t or p values, Odds ratios, beta coefficients)	**p**	**Sample size**
Cross-sectional association with PAct:						
If intervention/longitudinal: Association between baseline PAct and subsequent outcome:						
If intervention/longitudinal: Association between baseline PAct and change in outcome:						
If intervention/longitudinal: Association between change in PAct and subsequent outcome:						
If intervention/longitudinal: Association between change in PAct and change in outcome:						
Other/comments:						

To extract data for further outcomes, please copy and paste the table above and edit the "outcome" field.

Outcomes:

For peer review only

- systolic blood pressure
- diastolic blood pressure
- LDL/HDL/Total cholesterol
- serum triglycerides
- weight
- BMI
- Life expectancy/survival
- Diet
- Physical activity
- Smoking
- Alcohol
- Medication adherence

Mediation:

Only if intervention study. Add details of any formal mediation analyses to determine if PAct mediates intervention effects on outcomes.

Section 5: Conclusions

<u>**Conclusions**</u>	
**Author's conclusions:**	
**Limitations (e.g. multiplicity)**	
**Reviewer's conclusions/comments:**	

For peer review only

Reporting checklist for protocol of a systematic review and meta analysis.

Based on the PRISMA-P guidelines.

Instructions to authors

Complete this checklist by entering the page numbers from your manuscript where readers will find each of the items listed below.

Your article may not currently address all the items on the checklist. Please modify your text to include the missing information. If you are certain that an item does not apply, please write "n/a" and provide a short explanation.

Upload your completed checklist as an extra file when you submit to a journal.

In your methods section, say that you used the PRISMA-Preporting guidelines, and cite them as:

Moher D, Shamseer L, Clarke M, Ghersi D, Liberati A, Petticrew M, Shekelle P, Stewart LA. Preferred Reporting Items for Systematic Review and Meta-Analysis Protocols (PRISMA-P) 2015 statement. Syst Rev. 2015;4(1):1.

		Reporting Item	Page Number
**Title**			
Identification	#1a	Identify the report as a protocol of a systematic review	1
Update	#1b	If the protocol is for an update of a previous systematic review, identify as such	n/a
**Registration**			
	#2	If registered, provide the name of the registry (such as PROSPERO) and registration number	2
**Authors**			
Contact	#3a	Provide name, institutional affiliation, e-mail address of all protocol authors; provide physical mailing address of corresponding author	1
Contribution	#3b	Describe contributions of protocol authors and identify the guarantor of the review	18

For peer review only

**Amendments**			
	#4	If the protocol represents an amendment of a previously completed or published protocol, identify as such and list changes; otherwise, state plan for documenting important protocol amendments	17
**Support**			
Sources	#5a	Indicate sources of financial or other support for the review	18
Sponsor	#5b	Provide name for the review funder and / or sponsor	18
Role of sponsor or funder	#5c	Describe roles of funder(s), sponsor(s), and / or institution(s), if any, in developing the protocol	18
**Introduction**			
Rationale	#6	Describe the rationale for the review in the context of what is already known	3-5
Objectives	#7	Provide an explicit statement of the question(s) the review will address with reference to participants, interventions, comparators, and outcomes (PICO)	5
**Methods**			
Eligibility criteria	#8	Specify the study characteristics (such as PICO, study design, setting, time frame) and report characteristics (such as years considered, language, publication status) to be used as criteria for eligibility for the review	6-8
Information sources	#9	Describe all intended information sources (such as electronic databases, contact with study authors, trial registers or other grey literature sources) with planned dates of coverage	8
Search strategy	#10	Present draft of search strategy to be used for at least one electronic database, including planned limits, such that it could be repeated	8-9
Study records - data management	#11a	Describe the mechanism(s) that will be used to manage records and data throughout the review	10
Study records - selection process	#11b	State the process that will be used for selecting studies (such as two independent reviewers) through each phase of the review (that is, screening, eligibility and inclusion in meta-analysis)	10
Study records - data	#11c	Describe planned method of extracting data from reports (such as	9

For peer review only

collection process		piloting forms, done independently, in duplicate), any processes for obtaining and confirming data from investigators	
Data items	#12	List and define all variables for which data will be sought (such as PICO items, funding sources), any pre-planned data assumptions and simplifications	10; 25
Outcomes and prioritization	#13	List and define all outcomes for which data will be sought, including prioritization of main and additional outcomes, with rationale	10; 25
Risk of bias in individual studies	#14	Describe anticipated methods for assessing risk of bias of individual studies, including whether this will be done at the outcome or study level, or both; state how this information will be used in data synthesis	10
Data synthesis	#15a	Describe criteria under which study data will be quantitatively synthesised	15
Data synthesis	#15b	If data are appropriate for quantitative synthesis, describe planned summary measures, methods of handling data and methods of combining data from studies, including any planned exploration of consistency (such as I2, Kendall's τ)	15-16
Data synthesis	#15c	Describe any proposed additional analyses (such as sensitivity or subgroup analyses, meta-regression)	16
Data synthesis	#15d	If quantitative synthesis is not appropriate, describe the type of summary planned	14
Meta-bias(es)	#16	Specify any planned assessment of meta-bias(es) (such as publication bias across studies, selective reporting within studies)	16
Confidence in cumulative evidence	#17	Describe how the strength of the body of evidence will be assessed (such as GRADE)	11-14

The PRISMA-P elaboration and explanation paper is distributed under the terms of the Creative Commons Attribution License CC-BY. This checklist was completed on 07. August 2021 using https://www.goodreports.org/, a tool made by the EQUATOR Network in collaboration with Penelope.ai

For peer review only

The association between patient activation, self-management behaviours and clinical outcomes in adults with diabetes or related metabolic disorders: A systematic review and meta-analysis protocol

Journal:	*BMJ Open*
Manuscript ID	bmjopen-2021-056293.R1
Article Type:	Protocol
Date Submitted by the Author:	16-Dec-2021
Complete List of Authors:	Mueller, Julia; University of Cambridge, MRC Epidemiology Unit<br>Ahern, Amy; University of Cambridge, MRC Epidemiology Unit<br>Sharp, Stephen; University of Cambridge, MRC Epidemiology Unit<br>Richards, Rebecca; University of Cambridge, MRC Epidemiology Unit<br>Birch, Jack; University of Cambridge, MRC Epidemiology Unit<br>Davies, Alan; The University of Manchester, Division of Informatics, Imaging & Data Sciences<br>Griffin, Simon; University of Cambridge, MRC Epidemiology Unit; University of Cambridge, Department of Public Health and Primary Care
**Primary Subject Heading**:	Diabetes and endocrinology
Secondary Subject Heading:	Diabetes and endocrinology, Health services research
Keywords:	DIABETES & ENDOCRINOLOGY, HEALTH SERVICES ADMINISTRATION & MANAGEMENT, SOCIAL MEDICINE

SCHOLARONE™
Manuscripts

For peer review only - http://bmjopen.bmj.com/site/about/guidelines.xhtml

The association between patient activation, self-management behaviours and clinical outcomes in adults with diabetes or related metabolic disorders: A systematic review and meta-analysis protocol

For peer review only

*Julia Mueller**[1] julia.mueller@mrc-epid.cam.ac.uk

Amy L. Ahern[1] amy.ahern@mrc-epid.cam.ac.uk

Stephen J. Sharp[1] stephen.sharp@mrc-epid.cam.ac.uk

Rebecca Richards[1] rebecca.richards@mrc-epid.cam.ac.uk

Jack M. Birch[1] jack.birch@mrc-epid.cam.ac.uk

Alan Davies[2] alan.davies-2@manchester.ac.uk

Simon J. Griffin[1,3] profgp@medschl.cam.ac.uk

[1] MRC Epidemiology Unit, University of Cambridge, Cambridge, UK.

[2] Division of Informatics, Imaging & Data Sciences, University of Manchester, Manchester, Uk.

3 Department of Public Health and Primary Care, University of Cambridge, Cambridge, UK.

* Corresponding author: Julia Mueller, MRC Epidemiology Unit, University of Cambridge School of Clinical Medicine, Box 285 Institute of Metabolic Science, Cambridge Biomedical Campus, Cambridge, CB2 0QQ; Phone: +44 (0) 1223 769138

Keywords: Patient activation; diabetes; obesity; cardiovascular disease; self-management

Word count: 3823 words

For peer review only - http://bmjopen.bmj.com/site/about/guidelines.xhtml

Abstract

Introduction: Diabetes and related metabolic disorders such as obesity and cardiovascular diseases (CVD) are a growing global issue. Equipping individuals with the necessary 'knowledge, skills and confidence to self-manage their health' (i.e. patient activation [PAct]) may lead to improvements in health outcomes. It is unclear whether existing evidence allows us to assume a causal relationship. We aim to synthesise and critically appraise evidence on the relationship between PAct and self-management behaviours and clinical outcomes of people living with diabetes and related metabolic disorders.

Methods and analysis: The protocol is based on guidance on Preferred Reporting Items for Systematic Review and Meta-analysis Protocols (PRISMA-P). We will search Medline, Embase, CENTRAL, PsycInfo, Web of Science, and CINAHL using search terms related to patient activation, diabetes, prediabetes, obesity, and cardiovascular disease. Any quantitative study design is eligible provided studies assess the association between PAct and clinical outcomes and/or self-management behaviours of diabetes and related metabolic disorders. Outcomes include behavioural (e.g. diet) and clinical (e.g. blood pressure) outcomes. Two reviewers will independently screen titles/abstracts and full texts and assess risk of bias using the revised Cochrane risk-of-bias tool for randomized trials (RoB 2) or the Risk of Bias Assessment Tool for Nonrandomized Studies (RoBANS).

One reviewer will extract data, with independent checking by a second reviewer. We will critically assess the level of evidence available for assuming a causal association between PAct and outcomes. Data permitting, we will use the Hunter-Schmidt random-effects method to meta-analyse correlations across studies.

Ethics and dissemination: Ethical approval is not required. The review will be disseminated in the form of a peer-reviewed journal article, at conferences and other presentations. The findings of the review will be of interest to clinical commissioning groups, policy makers and intervention deliverers/developers.

Registration: Prospero registration number: CRD42021230727

For peer review only

Article Summary

Strengths and limitations of this study

- This review assesses whether patient activation is a proxy measure for wider health outcomes, and includes a broad range of clinical and behavioural outcomes
- It uses a comprehensive search strategy with a broad range of relevant databases, including databases that allow insight into grey literature (e.g. conference abstracts, theses)
- We will conduct a thorough critical appraisal of the evidence, based on a systematic procedure adapted from previous reviews, to assess whether evidence supports causal assumptions
- We expect high heterogeneity across studies, which may make meta-analysis infeasible or difficult to interpret

Background

Excess body weight is a major risk factor for chronic health problems such as diabetes mellitus and cardiovascular disease (CVD).[1,2] Diabetes and related metabolic disorders (e.g. obesity and CVD) are linked to poor patient outcomes such as reduced quality of life [e.g. 3] as well as increased direct and indirect economic costs, mainly due to medication, hospitalisations, disability and loss of productivity.[4–10] Equipping individuals with the necessary knowledge, skills and confidence to achieve sustained changes in their behaviour and self-manage their health and healthcare may lead to improvements in health-related outcomes and reduced hospitalisation and costs.[11–15]

The construct encompassing patients' knowledge, confidence and skills for self-management has been termed 'patient activation' (PAct).[16] Consumer driven health care approaches and many chronic illness care models assume that more "activated" patients (i.e. patients with the relevant knowledge, confidence and skills to self-manage their own health and healthcare) will play a more active role in managing their health and have better health outcomes [16]. Conversely, less "activated" patients are expected to be less likely to see out help, adhere to medical advice, and manage their own health. A recent systematic review on PAct in adults with chronic conditions identified two measures of PAct, the Patient Activation Measure (PAM) and Patient Assessment of Chronic Illness Care (PACIC), which includes a sub-domain on PAct.[17] The PAM is the most commonly used instrument to assess PAct. It is a self-report measure with either 22 or 13 items (short form).[16,18] PAM scores range from 0 to 100 with higher scores indicating higher activation.

For peer review only - http://bmjopen.bmj.com/site/about/guidelines.xhtml

PAM scores are categorised by four stages of activation: stage 1 (≤47.0) and stage 2 (47.1-55.1) are categorised as low activation levels, and stage 3 (55.2-67.0) and stage 4 (≥67.1) are categorised as high activation levels. The PAM is widely used in healthcare delivery and evaluation.[19,20] For example, within the UK National Health Service (NHS) the PAM is used for population segmentation and risk stratification in order to target and tailor interventions.[19] General Practitioner practices have used the PAM to tailor their diabetes review process such that participants with lower activation levels receive longer appointments than those with high activation levels.[20] PAM scores are also used to allocate different interventions to individuals with different activation levels. As such, it is important to understand how the PAM (and other PAct measures) are associated with clinical outcomes and self-management behaviours.

PAct and self-management behaviours relevant to diabetes and related metabolic disorders

There is some evidence to indicate that PAct is associated with self-reported self-management behaviours relevant to diabetes and related metabolic disorders, such as eating a healthy diet, being physically active, adhering to medication, and smoking cessation.[16,18,21–26] For some outcomes, such as self-reported physical activity, the relationship with PAct appears consistent.[16,18,21,22,24,25] For other outcomes the relationship is less clear. For example, some studies have found no significant association between PAct and smoking,[21–23] and in Hibbard & Tusler's study, correlations with diet-related variables (e.g. self-reported fruit and vegetable consumption) seemed to vary depending on the population and the specific behaviour measured.[22] Although several studies have assessed associations between PAct and self-management behaviours, this evidence has, to our knowledge, not been synthesised in a systematic review.

PAct and clinical outcomes of diabetes and related metabolic disorders

Self-reported behavioural measures are prone to error (which may be correlated with error in the measure of PAct) and bias. Furthermore, it is not clear how associations between PAct and health behaviours translate into clinical outcomes. As the PAM is used in the evaluation of healthcare systems and interventions,[19] it is important to understand not only if this measure (and any other PAct measures) predict self-management behaviours (such as adhering to a healthy diet), but also how PAct measures relate to clinical outcomes.

For peer review only - http://bmjopen.bmj.com/site/about/guidelines.xhtml

Several studies have found significant associations between PAct and clinical outcomes such as HbA_{1C}, blood glucose, triglycerides, cholesterol and blood pressure. [23,26–30] However, the evidence base is heterogeneous and complex, with some studies finding no significant associations,[26,28] significant associations opposite to those hypothesised,[26] or inconsistent patterns across PAct levels (i.e. unclear dose-response relationships).[27] The relationship between PAct and objective clinical outcomes is therefore unclear and warrants further investigation and synthesis.

PAct as a causal factor for health outcomes

The concept of PAct is often used to inform intervention development to support patient self-management and participation and engagement in health care.[19] The underlying assumption is that increases in PAct cause improvements in health outcomes. It is therefore important to understand not only whether there is an association between PAct and outcomes of diabetes and related metabolic disorders, but also whether there is evidence for a causal pathway.

Two systematic reviews have assessed the impact of interventions targeting PAct on diabetes outcomes and found some evidence for effects on glycaemic control and self-management behaviours.[31,32] However, many of the included interventions are complex and include several components, and formal mediation analyses to assess whether interventions effects were mediated by increases in PAct were not carried out. It is therefore difficult to ascertain whether interventions effected change through PAct or other mechanisms.

Findings from individual studies suggest PAct interventions can significantly decrease weight and blood pressure and improve glycaemic control in people with overweight or obesity,[33] as well as reducing risk factors for cardiovascular disease, such as smoking and lack of exercise.[34] However, to our knowledge, no systematic review has assessed the effects of PAct interventions for adults with overweight, obesity or CVD.

A systematic review of the literature is required to assess the association between PAct and outcomes of diabetes and related metabolic disorders, and to critically appraise the strength of this evidence.

Aims

The aims of this review are:

For peer review only - http://bmjopen.bmj.com/site/about/guidelines.xhtml

i. To systematically review and synthesise evidence on the association between PAct and self-management behaviours relevant to diabetes and related metabolic disorders (e.g. diet, physical activity).
ii. To systematically review and synthesise evidence on the association between PAct and clinical outcomes of diabetes and related metabolic disorders (e.g. blood pressure, HbA_{1c}).
iii. To critically appraise whether the evidence is sufficient to assume a causal role of PAct in improving clinical outcomes and self-management behaviours.

Methods

The protocol is based on guidance on conducting systematic reviews provided by the Centre for Reviews and Dissemination,[35] Preferred Reporting Items for Systematic Reviews and Meta-Analyses (PRISMA),[36] and Preferred Reporting Items for Systematic Review and Meta-analysis Protocols (PRISMA-P).[37]

We will adopt a 2-phase approach, whereby the first phase will involve a systematic scoping of the literature. This will involve establishing a list of all studies (cross-sectional, longitudinal, intervention) that examine the relationship between PAct and outcomes in our target population. Depending on the studies found in Phase 1, we will then consider whether we are able to narrow down our review questions, e.g. by population (e.g. only diabetes populations), or study design.

Inclusion/exclusion criteria

Studies will be eligible if they include a measure of PAct (e.g. PAM, PACIC) and assess the association between PAct and clinical outcomes and/or self-management behaviours relevant to diabetes and related metabolic disorders, or if they assess the effect on such outcomes of interventions that explicitly target patient activation.

Population

We will include studies with samples consisting of adults (≥ 18 years old) who have diabetes or a related metabolic disorder. We defined “diabetes and related metabolic disorders” to include prediabetes, diabetes (type 1/type 2 diabetes), obesity, and CVD. We define prediabetes as a state with glycaemic levels above ‘normal’ but below cut-offs for a diagnosis of diabetes. As such, we will include any studies that describe their population as being diagnosed with prediabetes, impaired glucose tolerance, glucose intolerance, impaired fasting glycaemia, borderline diabetes, non-diabetic hyperglycaemia, or similar.[38] We will not apply any specific criteria (e.g. cut-offs for impaired fasting glucose or impaired glucose tolerance). We define CVD as any conditions affecting the heart or blood vessels, including (but not limited to): coronary heart disease (angina, heart attacks, heart

For peer review only

failure), strokes and transient ischaemic attacks, peripheral arterial disease, and aortic disease. Studies will be eligible if they include one or more of these disease types in a broader sample if results are reported separately for our population of interest.

Interventions

We will include studies of varying designs, including intervention studies (see 'Study designs'). Where we include intervention studies, any type of intervention will be eligible as long as PAct is measured and the study reports on its association with our pre-defined outcomes, since the primary aim of the review is not to assess the effectiveness of a particular type of intervention but to assess the relationship between PAct and outcomes.

If an intervention study reports intervention effects on PAct and effects on other specified outcomes but does not report on the association between PAct and outcomes, we will include the study only if (i) the intervention explicitly aims to increase PAct or is described as targeting patients' knowledge, confidence and skills for self-management (as opposed to interventions that target related but different constructs such as self-efficacy) and (ii) increasing PAct is a key, main component of the intervention (i.e. studies will be excluded if PAct components form part of a complex intervention with other components). Such studies will be excluded from quantitative synthesis, but will be included in narrative synthesis as they can provide evidence of an association between PAct and outcomes.

Comparators

Where we include intervention studies, any type of comparator will be eligible (as well as observational studies or other intervention studies with no comparator, e.g. pre-post studies).

Exposure

We will include only studies that include a measure of PAct (e.g. PAM, PACIC, or other measures of PAct). We will not include studies that measure related constructs (e.g. confidence, or self-efficacy) if the measures do not explicitly purport to assess patient activation.

Outcomes

We will focus on clinical outcomes and self-management behaviours that are shared between diabetes and related metabolic disorders. Both self-reported and objectively measured outcomes will be eligible. We will include studies that measure at least one of the following outcomes:

Clinical outcomes

For peer review only - http://bmjopen.bmj.com/site/about/guidelines.xhtml

For peer review only

- HbA_{1C} level / glycaemic control
- Systolic blood pressure / diastolic blood pressure
- Low-density lipoprotein (LDL) / High-density lipoprotein (HDL) / Total cholesterol
- Serum triglycerides
- Body Mass Index (BMI) / body weight

Self-management behaviours

- Outcomes related to diet (e.g. fruit/vegetable consumption, following a low-fat diet)
- Outcomes related to physical activity (e.g. step counts, following a regular exercise schedule, frequency of physical activity)
- Outcomes related to smoking (e.g. smoking status)
- Outcomes related to alcohol consumption (e.g. alcohol consumption, frequency or amounts)
- Medication adherence

Study design

We will include original primary research articles. We will include all study designs, including cross-sectional, longitudinal and intervention (e.g. randomised controlled trials (RCTs), pre-post comparison studies) as long as studies report on the association between PAct and one of the specified outcomes. We will exclude study protocols, literature reviews/meta-analyses, qualitative studies, and studies not reporting on empirical data.

Language and date

We will include studies in any language, subject to local translation resources. Searches will not be limited by date.

Publication status

We will endeavour to include both published and unpublished materials (e.g. abstracts, theses) to reduce the impact of publication bias.[35]

Information sources and search strategy

Databases

The following databases will be searched:

- Medline
- Embase
- CENTRAL
- PsycInfo
- Web of Science
- CINAHL

For peer review only

Search strategy

The search strategy (Table 1) was devised with the help of a medical librarian. The search strategy is outlined in Table 1, and an example of the proposed search strategy is shown in Appendix A. References of included studies will be hand-searched for further eligible studies. Searches will be re-run prior to the final analysis. To identify relevant grey literature, we will search the Health Management Information Consortium (HMIC) database, ZETOC (using the conference search), and the British Library Integrated Catalogue.

Table 1. Search terms for the systematic review.

**Concept**	**Free text**	**MeSH**
Patient activation	“patient* activation*”<br>measure* ADJ5 “patient activation”<br>PAM?22*<br>PAM?13*<br>PAM??13*<br>PAM??22*<br>"Patient Assessment of Chronic Illness Care*"<br>PACIC*	
Diabetes	Diabet*<br>T2DM<br>T1DM<br>(non insulin* depend* or non insulin depend* or non insulin?depend* or non insulin?depend)<br>IDDM or NIDDM or MODY<br>T1D or T2D	exp Diabetes Mellitus, Type 2/ or exp Diabetes Mellitus/ or exp Diabetes Mellitus, Type 1<br><br>exp diabetes insipidus
Prediabetes	Pre?diabet*<br>Borderline ADJ3 diabet*<br>Impair* ADJ3 glucose<br>“Non-diabetic hyperglyc?emi*”<br>Glucose ADJ3 intoleran*	exp Prediabetic State/ or exp Glucose Intolerance/
Obesity/Overweight	Obes*<br>Overweight	exp Obesity/ OR

For peer review only - http://bmjopen.bmj.com/site/about/guidelines.xhtml

	"over weight"<br>Body ADJ3 weight<br>"body weight"<br>Adiposit*<br>Weight adj3 (gain* or loss* or chang* or control* or maintain* or reduc* or manag*)<br>Bmi or body mass ind*	exp Overweight/ OR<br>exp Body Weight/ OR<br>exp Adiposity/ or<br>exp body mass index/
Heart disease	Heart* OR<br>cardiovascular<br>OR<br>coronary OR cardio* OR cardiac*	exp Heart Diseases/ OR exp Cardiovascular Diseases/<br>exp Coronary Disease/ OR exp heart failure/

Data management and selection process

Citations returned through the database search will be exported into Covidence and de-duplicated for screening. Two reviewers will independently screen titles and abstracts for eligibility, and will then read full texts of selected citations to further assess eligibility. Any disagreements will be resolved by a third independent reviewer. Interrater reliability will be assessed using Cohen's Kappa.[39]

Data extraction

Initially, we will extract study information into a table to summarise broad study characteristics. We will use this to assess the available evidence and decide whether to narrow down our review objectives (e.g. to a specific disease population). Data from included studies will be extracted into a data extraction sheet (draft shown in Appendix B). The data extraction sheet is adapted from the Cochrane data collection form for RCTs and non-RCTs[40] and was also informed by the STROBE checklist of items that should be included in reports of observational studies,[41] the CONSORT statement,[42] and the risk of bias tools we used (Table 2).

Data to be extracted include details regarding study design, population, sample size, details about the intervention if relevant, methods used to assess outcomes, and details on the reported association between PAct and outcomes (including effect size, whether adjusted or unadjusted, and

For peer review only - http://bmjopen.bmj.com/site/about/guidelines.xhtml

For peer review only

what covariates were included in adjusted models). One reviewer will extract data and one reviewer will independently check this for accuracy and completeness. The data extraction sheet will be pilot-tested by at least 2 reviewers on three studies. Any issues will be discussed and the sheet will be updated accordingly.

Risk of bias / Quality appraisal

We will use two different tools to assess risk of bias, depending on study design (Table 2).

Table 2. Risk of bias tools to be used in the review, depending on study design.

**Study design**	**Risk of bias tool**
Randomised controlled trial*	RoB 2: A revised Cochrane risk-of-bias tool for randomized trials[43]
Observational studies	Risk of Bias Assessment Tool for Nonrandomized Studies (RoBANS)[44]

** RCTs that have been analysed as a cohort study (i.e. reporting on the association between PAct and outcomes, regardless of study group allocation), will be assessed using the RoBANS tool. If the data we extract depend on study group allocation, we will use the RoB 2 tool.*

Each study will be appraised by two independent review authors. Reviewers will discuss any discrepancies until they reach a consensus, consulting a third reviewer if required. Any potential sources of bias or methodological limitations not covered by the tools will be noted by the reviewers. Each study will be assigned an overall risk rating of high, low or unclear (RoBANS tool) or high/low/some concerns (ROB 2). Risk of bias assessments will be used to determine the level of evidence (see section on 'Levels of evidence'). For the purpose of determining the level of evidence, risk of bias will be dichotomised into high/low risk (for RoBANS, 'unclear' and 'high' and for ROB2, 'some concerns' and 'high' will be amalgamated).

Data synthesis and analysis

The study selection process will be depicted in a PRISMA diagram. Key results will be presented in form of a table summarising study characteristics. Risk of bias assessments will also be provided in a table.

Narrative synthesis: Levels of evidence

A key output of this review will be an assessment of the level of evidence available for assuming a causal association between PAct and self-management behaviours as well as clinical outcomes of diabetes and related metabolic disorders. The 'level of evidence' will be a composite measure, based

For peer review only - http://bmjopen.bmj.com/site/about/guidelines.xhtml

on the strength of the study design/analysis, the quality of the study, sample size, and the consistency of the findings, adapted from an approach used in a previous systematic review.[45]

Table 3shows the types of study designs, coupled with different types of analyses, that could provide evidence for a causal assumption, grouped into different categories based on their suitability to support this assumption. If we encounter any unanticipated study designs/analyses, we will discuss this within the review team to assign the appropriate categorisation.

Once study designs and analyses have been categorised according to Table 3 and once studies have been assigned a risk of bias appraisal, we will use Figure 1 and Figure 2 to assign a level of evidence, depending on the consistency of the findings across studies. Findings will be considered to be consistent if at least two thirds (66.6%) of the highest quality studies are reported to have significant results in the same direction.[45]

Table 3. Categorisation of the suitability of different study designs (coupled with different analyses) to draw conclusions regarding a causal association between PAct and outcomes of diabetes and related metabolic disorders. PAct = patient activation

**Possible study designs + analyses**	**Suitability of study design and analyses**	**Rationale**
RCTs with causal mediation analysis to assess whether PAct mediates intervention effects	strong	RCTs are the only study design that allow causal mediation analysis to identify the mechanisms by which interventions exert their effects[46]
Cohort studies / RCTs or other intervention studies that assess the association between PAct and subsequent outcomes	moderate	RCTs and longitudinal observational studies can provide temporal insights into the association between PAct and outcomes, which gives some indication of causality.[47] If an RCT examines the association between PAct and outcomes independent of study group allocation, randomisation has no bearing; analyses & findings are therefore akin to cohort studies.
RCTs that do not report on the association between PAct and outcomes but that show intervention effects on outcomes AND intervention effects on PAct, AND the intervention explicitly, mainly addresses PAct	moderate	RCTs provide insight into causal effects of interventions on outcomes. If an intervention explicitly addresses PAct and there is evidence that the intervention influenced both PAct and outcomes, this provides indication for a causal mechanism of PAct on outcomes (though not definitive).
Observational cross-sectional studies	weak	In cross-sectional designs, the time order of effects cannot be determined and therefore causality cannot be inferred.[48]

For peer review only - http://bmjopen.bmj.com/site/about/guidelines.xhtml

Intervention studies that are not RCTs (e.g. pre-post studies) and that do not report on the association between PAct and outcomes but that show changes in outcomes AND changes in PAct.	weak	Pre-post designs have the strength of temporality to indicate outcomes might be impacted by an intervention, but due to lack of randomisation causality cannot be inferred.[49]

For peer review only

For peer review only - http://bmjopen.bmj.com/site/about/guidelines.xhtml

[Insert Figure 1 and 2 here]

Narrative synthesis: Harvest Plot

If meta-analysis is not feasible and we cannot produce forest plots, we will create Harvest Plots to synthesise and depict our findings, adapted from the approach used by Ogilvie et al.[50] The plot will consist of a matrix with one row per outcome, and one column (for the assumption that there is a causal relationship between PAct and outcomes). Each study will be represented by a bar in each row for which that study reported relevant evidence. The strength of the study design and the analysis will be represented by the height of the bar, with higher bars indicating more suitable design and analysis. Studies using self-reported outcomes will be represented by a grey bar, while bars for studies using objective measures will be black. Each bar will be annotated with the quality appraisal for that study (e.g. high, low or unclear) and the sample size.

Meta-analysis

Meta-analysis will be undertaken if studies are considered sufficiently similar in their research questions, designs and outcomes. From each study, we will extract effect sizes for the association between PAct and the pre-specified outcomes. We will extract unadjusted and adjusted associations, and synthesise these separately. Regression coefficients from models with different sets of covariates represent different parameters and cannot be combined meaningfully.[51] We will therefore initially assess which covariates are included in adjusted models and, if there is agreement between models in terms of key covariates, we will synthesise coefficients across models (even if model specifications are not completely identical). If there is insufficient agreement between models in terms of covariates, we will include adjusted associations in the narrative synthesis, and focus on unadjusted associations in the quantitative synthesis.

We expect studies to report a wide range of different estimates of the association between PAct and outcomes. We will therefore initially convert different measures of the association to the Pearson Product Moment Correlation using the formulae in Table 4, because the correlation coefficient is an easily interpretable effect size to assess the strength of association between two variables. Some studies may report only odds ratios (as PAct scores are often dichotomised into high/low and clinical outcomes are often dichotomised into within/not within normal range). If studies report odds ratios, we will construct contingency tables based on information about percentages of PAct levels and outcomes and use these tables to calculate χ^2 values, which can then be transformed to r.

We will use a random-effects approach, because we assume that the population effect sizes vary randomly from study to study (rather than assuming the population effect size is the same for all

For peer review only - http://bmjopen.bmj.com/site/about/guidelines.xhtml

studies), e.g. due to differences in age, socioeconomic status, geographic location, or disease. Random effects meta-analysis allows inferences beyond the studies included in the analysis.[52] However, if the number of included studies is ≤5, we will also perform a sensitivity analysis with a fixed-effect approach. This is because when heterogeneity is present, a random-effects meta-analysis weights the studies relatively more equally than a fixed-effect analysis, and thus small-study effects could bias the findings.

We will use the Hunter-Schmidt random-effects method to synthesise correlations across studies, because this method produces more accurate estimates than the Hedges-Olkin and Rosenthal-Rubin methods (except when the average population effect size is very large).[52] Effect sizes from cross-sectional and longitudinal studies will be synthesised separately.

If a study reports more than one estimate of association for a particular combination of exposure and outcome, we will select the estimated association based on the longest duration of follow-up or the most precise measure of the outcome. If it is not possible to discern this, within-study meta-analytic calculations will be used to obtain a single effect size, to maintain the statistical assumption of independence necessary for a meta-analysis. If the effect sizes are based on different sample sizes, the average sample size will be calculated and used for subsequent analyses. **Error! Reference source not found.**

Table 4. Formulae to convert different measures of effect to Pearson's r, based on Wolf (1986),[53] *Friedman (1982),*[54] *and Hoeve at al. (2009)*[55]

Statistic to be converted	Formula for transforming to Pearson Product Moment Correlation r	Notes
T	$\sqrt{\frac{t^2}{t^2 + df}}$	
F(df=1)	$\sqrt{\frac{F}{F + df_D}}$	Use only for comparing two group means (df=1)<br>df_D: df of the denominator
F(df>1)	$\sqrt{\frac{df_N(F-1)}{df_N + df_D}}$	df_N: df of the numerator (k-1)<br>df_D: df of the denominator (N-k)
χ^2 (df=1)	$\sqrt{\frac{\chi^2}{n}}$	Use only for 2x2 frequency tables (df=1)
χ^2 (df>1)	$\sqrt{\frac{\chi^2}{\chi^2 + N}}$	
D	$\sqrt{\frac{d}{d^2 + 4}}$	
Φ	(1) $\chi^2 = \phi^2 * N$<br>(2) Use equation for χ^2(df=1) or χ^2 (df>1)	

For peer review only - http://bmjopen.bmj.com/site/about/guidelines.xhtml

Exploration of heterogeneity

If sufficient studies are available, we will perform meta-regression to assess whether the effect size varies with study characteristics, including:

- Studies with different populations (diabetes/prediabetes, obesity, CVD)
- Self-reported vs. objectively measured outcomes
- Clinical vs. behavioural outcomes

Meta-regression will be performed on correlations transformed according to the Fisher z-transformation.[56]

Sensitivity analyses

Sensitivity analysis will be performed excluding studies that are categorised as high risk of bias, to assess whether findings are unduly influenced by these studies.

Assessment of heterogeneity and reporting bias

To assess heterogeneity, we will report the I^2 statistic with a 95% confidence interval, as well as outcomes from the test for heterogeneity (Q-statistic and associated p-value). For I^2, we will categorise heterogeneity as low (0%–30%), moderate (30%–60%), substantial (60%–90%) and considerable (90%–100%).[57] To assess publication bias, we will construct funnel plots, plotting the mean correlation against study sample sizes as well as the residual standard deviation of r against the sample size.

Patient and Public Involvement

We shared a lay summary of the review protocol with an established patient and public involvement (PPI) panel. Feedback was positive, with panel members commenting that they think the review will be useful, particularly within NHS services. Panel members also made recommendations for our dissemination strategy to help us reach a wider audience. After completing the review, we will seek feedback from the PPI panel on a lay summary of the review findings and on our dissemination plan. The protocol was further reviewed by a GP partner from NHS Cambridgeshire and Peterborough CCG, who has particular expertise in person centred, collaborative care and long-term conditions.

Ethics and dissemination

Ethical approval is not required for this systematic review. The review will be disseminated in the form of a peer-reviewed journal article, at conferences and other presentations (e.g. webinars), as

For peer review only - http://bmjopen.bmj.com/site/about/guidelines.xhtml

well as more publicly accessible formats such as blog posts, social media posts, and, if suitable, a press release. The findings of the review will be of interest to clinical commissioning groups, policy makers and intervention deliverers/developers that currently use, or plan to use, the PAM or other measures of PAct to tailor and allocate interventions for diabetes and related metabolic disorders. It will also be of relevance to those using measures of PAct to evaluate intervention effectiveness and healthcare performance, as it will provide an indication of how well PAct predicts outcomes for diabetes and related metabolic disorders.

Amendments

Amendments made will be noted in a pre-specified section of the protocol (rather than being incorporated into the protocol), with the date and rationale. Amendments will also be uploaded to Prospero. Since commencing title/abstract screening, we have made one amendment (Table 5).

Table 5. Amendments to the protocol.

Date	Change	Rationale
29/01/2021	Removed "Life expectancy/ total survival" from the list of outcomes	After discussion within the team, we decided this outcome does not align well with the other included outcomes. The other outcomes give an indication of how well people self-manage their condition, whereas life expectancy/survival is a wider measure that gives less insight into self-management specifically. Moreover, there are unlikely to be many studies with sufficiently long follow-up to provide any meaningful assessment of survival in this context, and even if there was a study with very long follow-up, we would then be relying on an assumption that the patient activation exposures measured at baseline do not change over time.

Author contributions

JM drafted the manuscript, with regular input from all co-authors. All authors read, provided feedback and approved the manuscript prior to submission. JM, AA, SG, RR, JB and AD contributed to the development of the selection criteria, the risk of bias assessment strategy and data extraction criteria. SS provided statistical expertise.

For peer review only - http://bmjopen.bmj.com/site/about/guidelines.xhtml

Funding statement

This work was supported by the Medical Research Council [grant number MC_UU_00006/6] and the National Institute for Health Research (NIHR) under its Programme Grants for Applied Research Programme (RP-PG-0216-20010).

Competing interests statement

JM and RR are Trustees for the Association of the Study of Obesity (unpaid roles). ALA and SJG are the chief investigators on two publicly funded (MRC, NIHR) trials where the intervention is provided by WW (formerly Weight Watchers) at no cost outside the submitted work. All other authors report no competing interests.

Acknowledgements

We would like to thank Dr Isla Kuhn for reviewing our protocol and helping us refine our search strategy. We also thank our PPI panel and Dr Mark Brookes for reviewing our protocol and providing feedback and comments.

References

1 Van Gaal LF, Mertens IL, De Block CE. Mechanisms linking obesity with cardiovascular disease. Nature. 2006;**444**:875–80. doi:10.1038/nature05487

2 Al-Goblan AS, Al-Alfi MA, Khan MZ. Mechanism linking diabetes mellitus and obesity. *Diabetes, Metab Syndr Obes Targets Ther* 2014;**7**:587–91. doi:10.2147/DMSO.S67400

3 Speight J, Holmes-Truscott E, Hendrieckx C, *et al.* Assessing the impact of diabetes on quality of life: what have the past 25 years taught us? *Diabet Med* 2020;**37**:dme.14196. doi:10.1111/dme.14196

4 Mata-Cases M, Casajuana M, Franch-Nadal J, *et al.* Direct medical costs attributable to type 2 diabetes mellitus: a population-based study in Catalonia, Spain. *Eur J Heal Econ* 2016;**17**:1001–10. doi:10.1007/s10198-015-0742-5

5 Giorda CB, Rossi MC, Ozzello O, *et al.* Healthcare resource use, direct and indirect costs of hypoglycemia in type 1 and type 2 diabetes, and nationwide projections. Results of the HYPOS-1 study. *Nutr Metab Cardiovasc Dis* 2017;**27**:209–16.

For peer review only - http://bmjopen.bmj.com/site/about/guidelines.xhtml

doi:10.1016/j.numecd.2016.10.005

6 Bain SC, Bekker Hansen B, Hunt B, *et al.* Evaluating the burden of poor glycemic control associated with therapeutic inertia in patients with type 2 diabetes in the UK. *J Med Econ* 2020;**23**:98–105. doi:10.1080/13696998.2019.1645018

7 Einarson TR, Acs A, Ludwig C, *et al.* Economic Burden of Cardiovascular Disease in Type 2 Diabetes: A Systematic Review. Value Heal. 2018;**21**:881–90. doi:10.1016/j.jval.2017.12.019

8 Tremmel M, Gerdtham UG, Nilsson PM, *et al.* Economic burden of obesity: A systematic literature review. Int. J. Environ. Res. Public Health. 2017;**14**. doi:10.3390/ijerph14040435

9 Bächle C, Claessen H, Andrich S, *et al.* Direct costs in impaired glucose regulation: results from the population-based Heinz Nixdorf Recall study. *BMJ Open Diabetes Res Care* 2016;**4**:e000172. doi:10.1136/BMJDRC-2015-000172

10 Ryder S, Fox K, Rane P, *et al.* A Systematic Review of Direct Cardiovascular Event Costs: An International Perspective. *PharmacoEconomics 2019 377* 2019;**37**:895–919. doi:10.1007/S40273-019-00795-4

11 Tay JHT, Jiang Y, Hong J, *et al.* Effectiveness of lay-led, group-based self-management interventions to improve glycated hemoglobin (HbA1c), self-efficacy, and emergency visit rates among adults with type 2 diabetes: A systematic review and meta-analysis. *Int J Nurs Stud* 2020;:103779. doi:10.1016/j.ijnurstu.2020.103779

12 Zhao Q, Chen C, Zhang J, *et al.* Effects of self-management interventions on heart failure: Systematic review and meta-analysis of randomized controlled trials. Int. J. Nurs. Stud. 2020;**110**:103689. doi:10.1016/j.ijnurstu.2020.103689

13 Newman S, Steed L, Mulligan K. Self-management interventions for chronic illness. In: *Lancet*. Elsevier 2004. 1523–37. doi:10.1016/S0140-6736(04)17277-2

14 Galani C, Schneider H. Prevention and treatment of obesity with lifestyle interventions: Review and meta-analysis. Int. J. Public Health. 2007;**52**:348–59. doi:10.1007/s00038-007-7015-8

15 Zhang D, Cogswell ME, Wang G, *et al.* Evidence of Dietary Improvement and Preventable Costs of Cardiovascular Disease. Am. J. Cardiol. 2017;**120**:1681–8. doi:10.1016/j.amjcard.2017.07.068

16 Hibbard JH, Stockard J, Mahoney ER, *et al.* Development of the Patient Activation Measure

For peer review only - http://bmjopen.bmj.com/site/about/guidelines.xhtml

(PAM): Conceptualizing and Measuring Activation in Patients and Consumers. *Health Serv Res* 2004;**39**:1005–26. doi:10.1111/j.1475-6773.2004.00269.x

17 Newland P, Lorenz R, Oliver BJ. Patient activation in adults with chronic conditions: A systematic review. *J Health Psychol* Published Online First: 2020. doi:10.1177/1359105320947790

18 Hibbard JH, Mahoney ER, Stockard J, *et al.* Development and testing of a short form of the patient activation measure. *Health Serv Res* 2005;**40**:1918–30. doi:10.1111/j.1475-6773.2005.00438.x

19 Hibbard J, Gilburt H. Supporting people to manage their health: An introduction to patient activation. 2014. https://www.kingsfund.org.uk/sites/default/files/field/field_publication_file/supporting-people-manage-health-patient-activation-may14.pdf

20 NHS England. Patient activation and PAM FAQs. https://www.england.nhs.uk/personalisedcare/supported-self-management/patient-activation/pa-faqs/ (accessed 9 Sep 2020).

21 Rask KJ, Ziemer DC, Kohler SA, *et al.* Patient activation is associated with healthy behaviors and ease in managing diabetes in an indigent population. *Diabetes Educ* 2009;**35**:622–30. doi:10.1177/0145721709335004

22 Hibbard JH, Tusler M. Assessing activation stage and employing a 'next steps' approach to supporting patient self-management. *J Ambul Care Manage* 2007;**30**:2–8. doi:10.1097/00004479-200701000-00002

23 Hendriks M, Rademakers J. Relationships between patient activation, disease-specific knowledge and health outcomes among people with diabetes; a survey study. *BMC Health Serv Res* 2014;**14**:393. doi:10.1186/1472-6963-14-393

24 Harvey L, Fowles JB, Xi M, *et al.* When activation changes, what else changes? The relationship between change in patient activation measure (PAM) and employees' health status and health behaviors. *Patient Educ Couns* 2012;**88**:338–43. doi:10.1016/j.pec.2012.02.005

25 Hibbard JH, Mahoney ER, Stock R, *et al.* Do Increases in Patient Activation Result in Improved Self-Management Behaviors? *Health Serv Res* 2007;**42**:1443–63. doi:10.1111/j.1475-6773.2006.00669.x

For peer review only - http://bmjopen.bmj.com/site/about/guidelines.xhtml

26 Greene J, Hibbard JH. Why does patient activation matter? An examination of the relationships between patient activation and health-related outcomes. *J Gen Intern Med* 2012;**27**:520–6. doi:10.1007/s11606-011-1931-2

27 Sacks RM, Greene J, Hibbard J, *et al.* Does patient activation predict the course of type 2 diabetes? A longitudinal study. *Patient Educ Couns* 2017;**100**:1268–75. doi:10.1016/j.pec.2017.01.014

28 Woodard LCD, Landrum CR, Amspoker AB, *et al.* Interaction between functional health literacy, patient activation, and glycemic control. Patient Prefer. Adherence. 2014;**8**:1019–24. doi:10.2147/PPA.S63954

29 Rogvi S, Tapager I, Almdal TP, *et al.* Patient factors and glycaemic control - associations and explanatory power. *Diabet Med* 2012;**29**. doi:10.1111/j.1464-5491.2012.03703.x

30 Remmers C, Hibbard J, Mosen DM, *et al.* Is patient activation associated with future health outcomes and healthcare utilization among patients with diabetes? *J Ambul Care Manage* 2009;**32**:320–7. doi:10.1097/JAC.0b013e3181ba6e77

31 Bolen SD, Chandar A, Falck-Ytter C, *et al.* Effectiveness and safety of patient activation interventions for adults with type 2 diabetes: Systematic review, meta-analysis, and meta-regression. J. Gen. Intern. Med. 2014;**29**:1166–76. doi:10.1007/s11606-014-2855-4

32 Almutairi N, Hosseinzadeh H, Gopaldasani V. The effectiveness of patient activation intervention on type 2 diabetes mellitus glycemic control and self-management behaviors: A systematic review of RCTs. Prim. Care Diabetes. 2020;**14**:12–20. doi:10.1016/j.pcd.2019.08.009

33 Barnason S, Zimmerman L, Schulz P, *et al.* Weight management telehealth intervention for overweight and obese rural cardiac rehabilitation participants: A randomised trial. *J Clin Nurs* 2019;**28**:1808–18. doi:10.1111/jocn.14784

34 Tinsel I, Siegel A, Schmoor C, *et al.* Encouraging Self-Management in Cardiovascular Disease Prevention. *Dtsch Arztebl Int* 2018;**115**:469–76. doi:10.3238/arztebl.2018.0469

35 Centre for Reviews and Dissemination. Systematic Reviews: CRD's guidance for undertaking reviews in health care. 2009.

36 Liberati A, Altman DG, Tetzlaff J, *et al.* The PRISMA statement for reporting systematic reviews and meta-analyses of studies that evaluate healthcare interventions: explanation and elaboration. *BMJ* 2009;**339**:b2700. doi:10.1136/bmj.b2700

For peer review only - http://bmjopen.bmj.com/site/about/guidelines.xhtml

37 Moher D, Shamseer L, Clarke M, *et al.* Preferred reporting items for systematic review and meta-analysis protocols (PRISMA-P) 2015 statement. *Syst Rev 2015 41* 2015;**4**:1–9. doi:10.1186/2046-4053-4-1

38 Diabetes UK. Prediabetes. https://www.diabetes.org.uk/preventing-type-2-diabetes/prediabetes (accessed 6 Jan 2021).

39 Cohen J. A Coefficient of Agreement for Nominal Scales. *Educ Psychol Meas* 1960;**20**:37–46. doi:10.1177/001316446002000104

40 The Cochrane Collaboration. Data extraction forms. 2020.https://dplp.cochrane.org/data-extraction-forms

41 STROBE Statement. STROBE checklists. https://www.strobe-statement.org/index.php?id=available-checklists (accessed 16 Oct 2020).

42 Schulz KF, Altman DG, Moher D. CONSORT 2010 Statement: Updated guidelines for reporting parallel group randomised trials. *BMJ* 2010;**340**:698–702. doi:10.1136/bmj.c332

43 Sterne JAC, Savović J, Page MJ, *et al.* RoB 2: A revised tool for assessing risk of bias in randomised trials. *BMJ* 2019;**366**. doi:10.1136/bmj.l4898

44 Kim SY, Park JE, Lee YJ, *et al.* Testing a tool for assessing the risk of bias for nonrandomized studies showed moderate reliability and promising validity. *J Clin Epidemiol* 2013;**66**:408–14. doi:10.1016/j.jclinepi.2012.09.016

45 Van Sluijs EMF, McMinn AM, Griffin SJ. Effectiveness of interventions to promote physical activity in children and adolescents: Systematic review of controlled trials. *Br J Sports Med* 2008;**42**:653–7. doi:10.1136/bmj.39320.843947.BE

46 Lee H, Herbert RD, Lamb SE, *et al.* Investigating causal mechanisms in randomised controlled trials. *Trials* 2019;**20**:524. doi:10.1186/s13063-019-3593-z

47 Barnett ML, Hyman JJ. Challenges in interpreting study results The conflict between appearance and reality. 2006. doi:10.14219/jada.archive.2006.0405

48 Porta M. *Dictionary of Epidemiology*. Oxford: : Oxford University Press 2008.

49 Thiese MS. Observational and interventional study design types; an overview. *Biochem Medica* 2014;**24**:199–210. doi:10.11613/BM.2014.022

50 Ogilvie D, Fayter D, Petticrew M, *et al.* The harvest plot: A method for synthesising evidence

For peer review only - http://bmjopen.bmj.com/site/about/guidelines.xhtml

about the differential effects of interventions. *BMC Med Res Methodol* 2008;**8**:8. doi:10.1186/1471-2288-8-8

51 Aloe AM. Inaccuracy of regression results in replacing bivariate correlations. *Res Synth Methods* 2015;**6**:21–7. doi:10.1002/jrsm.1126

52 Field AP. Meta-analysis of correlation coefficients: A Monte Carlo comparison of fixed- and random-effects methods. *Psychol Methods* 2001;**6**:161–80. doi:10.1037/1082-989X.6.2.161

53 Wolf F. *Meta-Analysis*. 2455 Teller Road, Newbury Park California 91320 United States of America : : SAGE Publications, Inc. 1986. doi:10.4135/9781412984980

54 Friedman H. Simplified Determinations of Statistical Power, Magnitude of Effect and Research Sample Sizes. *Educ Psychol Meas* 1982;**42**:521–6. doi:10.1177/001316448204200214

55 Hoeve M, Dubas JS, Eichelsheim VI, *et al.* The relationship between parenting and delinquency: A meta-analysis. J. Abnorm. Child Psychol. 2009;**37**:749–75. doi:10.1007/s10802-009-9310-8

56 Dingman HF, Perry NC. A Comparison of the Accuracy of the Formula for the Standard Error of Pearson “r” with the accuracy of Fisher’s z-Transformation. *J Exp Educ* 1956;**24**:319–21. doi:10.1080/00220973.1956.11010555

57 Ryan R, Cochrane Consumers and Communication Review Group. Heterogeneity and subgroup analyses in Cochrane consumers and communication group reviews: planning the analysis at protocol stage. 2016.http://cccrg.cochrane.org (accessed 8 Jun 2020).

For peer review only

For peer review only - http://bmjopen.bmj.com/site/about/guidelines.xhtml

Figure Legends

Figure 1. Levels of evidence (part 1). To be used in conjunction with Table 3 and Figure 2. Note: studies including ≤ 250 participants or studies not providing sample size justifying a smaller sample size are considered 'small', studies including >250 participants are considered 'large'. Findings are considered consistent if at least two thirds (66.6%) of the highest quality studies are reported to have significant results in the same direction.

Figure 2. Levels of evidence (part 2). To be used in conjunction with Table 3 And Figure 1. Note: studies including ≤ 250 participants or studies not providing sample size justifying a smaller sample size are considered 'small', studies including >250 participants are considered 'large'. Findings are considered consistent if at least two thirds (66.6%) of the highest quality studies are reported to have significant results in the same direction.

For peer review only

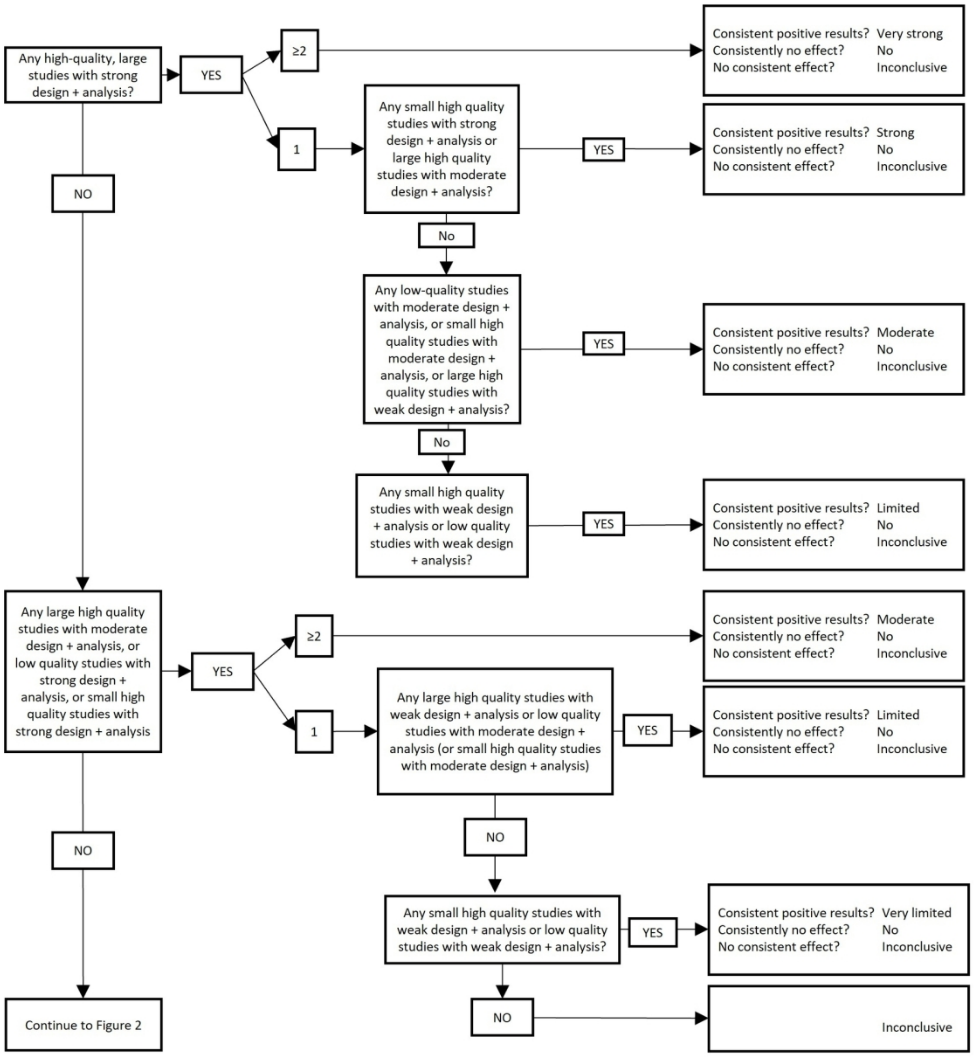

Figure 1. Levels of evidence (part 1). To be used in conjunction with Table 3 and Figure 2. Note: studies including ≤ 250 participants or studies not providing sample size justifying a smaller sample size are considered 'small', studies including >250 participants are considered 'large'. Findings are considered consistent if at least two thirds (66.6%) of the highest quality studies are reported to have significant results in the same direction.

154x176mm (300 x 300 DPI)

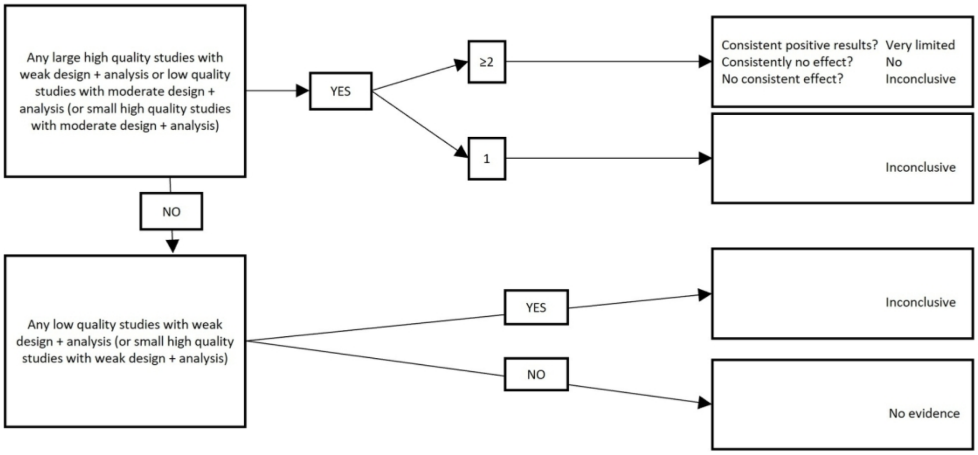

Figure 2. Levels of evidence (part 2). To be used in conjunction with Table 3 And Figure 1. Note: studies including ≤ 250 participants or studies not providing sample size justifying a smaller sample size are considered ‘small’, studies including >250 participants are considered ‘large’. Findings are considered consistent if at least two thirds (66.6%) of the highest quality studies are reported to have significant results in the same direction.

154x90mm (300 x 300 DPI)

Appendix A: Example search strategy

Medline (Ovid)

1	("patient* activation*" or (measure* adj5 "patient activation") or PAM?22* or PAM?13* or PAM??13* or PAM??22* or "Patient Assessment of Chronic Illness Care*" or PACIC*).mp. [mp=title, abstract, original title, name of substance word, subject heading word, floating sub-heading word, keyword heading word, organism supplementary concept word, protocol supplementary concept word, rare disease supplementary concept word, unique identifier, synonyms]
2	(Diabet* or T2DM or T1DM or (non insulin* depend* or non insulin depend* or non insulin?depend* or non insulin?depend) or IDDM or NIDDM or MODY or T1D or T2D).mp. or exp Diabetes Mellitus, Type 2/ or exp Diabetes Mellitus/ or exp Diabetes Mellitus, Type 1/ or exp diabetes insipidus/ or exp Diabetes, Gestational/ [mp=title, abstract, original title, name of substance word, subject heading word, floating sub-heading word, keyword heading word, organism supplementary concept word, protocol supplementary concept word, rare disease supplementary concept word, unique identifier, synonyms]
3	(Pre?diabet* or (Borderline adj3 diabet*) or (Impair* adj3 glucose) or (Non-diabetic adj3 hyperglyc?emi*) or (Glucose adj3 intoleran*)).mp. or exp Prediabetic State/ or exp Glucose Intolerance/ [mp=title, abstract, original title, name of substance word, subject heading word, floating sub-heading word, keyword heading word, organism supplementary concept word, protocol supplementary concept word, rare disease supplementary concept word, unique identifier, synonyms]
4	(Obes* or Overweight or "over weight" or (Body adj3 weight) or "body weight" or Adiposit* or (Weight adj3 (gain* or loss* or chang* or control* or maintain* or reduc* or manag*)) or Bmi or body mass ind*).mp. or exp Obesity/ or exp Overweight/ or exp Body Weight/ or exp Adiposity/ or exp body mass index/ [mp=title, abstract, original title, name of substance word, subject heading word, floating sub-heading word, keyword heading word, organism supplementary concept word, protocol supplementary concept word, rare disease supplementary concept word, unique identifier, synonyms]
5	(Heart* or cardiovascular or coronary or cardio* or cardiac*).mp. or exp Heart Diseases/ or exp Cardiovascular Diseases/ or exp Coronary Disease/ or exp Heart Failure/ [mp=title, abstract, original title, name of substance word, subject heading word, floating sub-heading word, keyword heading word, organism supplementary concept word, protocol supplementary concept word, rare disease supplementary concept word, unique identifier, synonyms]
6	2 or 3 or 4 or 5
7	1 and 6

For peer review only

For peer review only - http://bmjopen.bmj.com/site/about/guidelines.xhtml

Appendix B: Data extraction sheet

Section 1: General meta-data

**Review title**	The association between patient activation, self-management behaviours and clinical outcomes in diabetes and related metabolic disorders: A systematic review					
**Study ID** *(surname of first author and year first full report of study was published e.g. Smith 2001)*						
**Date form completed** *(dd/mm/yyyy)*						
**Initials of person extracting data:**						
**Title:**						
**Author(s):**						
**Source:**						
**Date:**		**Vol:**		**Issue:**		**Pages:**
**Publication type** (e.g. full report, abstract)						

Section 2: Study eligibility

Study characteristics	Eligibility criteria	Eligibility criteria met?		
		Yes	No	Un-clear
Population	Adults (≥ 18 years old) with diabetes or a related metabolic disorder (prediabetes, type 1 and type 2 diabetes , obesity, or CVD)	☐	☐	☐
Exposure	Includes a measure of patient activation (PAct)	☐	☐	☐
Outcomes	Includes at least one of the predefined outcomes, either clinical outcomes (HbA1C level/ glycaemic control, systolic blood pressure, diastolic blood pressure, low-density lipoprotein (LDL), high-density lipoprotein (HDL), total cholesterol, serum	☐	☐	☐

For peer review only - http://bmjopen.bmj.com/site/about/guidelines.xhtml

	triglycerides, BMI / weight, life expectancy/survival) or self-management behaviours (diet, physical activity, smoking, alcohol, medication adherence)			
Type of study	Original, primary research articles	☐	☐	☐
	Assesses the relationship between PAct and at least one of the defined outcomes	☐	☐	☐
	If no to the above: Is it an intervention study that reports intervention effects on PAct and effects on other specified outcomes AND (i) the intervention explicitly aims to increase patient activation or is described as targeting patients' knowledge, confidence and skills for self-management AND (ii) increasing patient activation is the main component of the intervention	☐	☐	☐
INCLUDE ☐		EXCLUDE ☐		
Reason for exclusion:				

For peer review only

DO NOT PROCEED IF STUDY EXCLUDED FROM REVIEW

Section 3: Objectives and design

**Objective:**	
**Setting:**	
**Country of origin:**	
**Start and end date:**	
**Study design**	
**Study population:**	
**Recruitment methods:**	
**Inclusion and exclusion criteria for participants:**	
**Sample size:**	

**Is a justification for the sample size provided (power calculation)?**	Yes/No (delete as appropriate)<br><br>Details:
**Withdrawals and exclusions:**	
**Attrition (i.e. loss to follow-up):**<br><br>**(For intervention studies, report per study group)**	

Section 2: Intervention details

Only complete Section 2 if it is an intervention study and we are interested in findings that depend on study group allocation. If it is an observational study, or an intervention study but the relevant data to extract pertain to the association between PAct and outcomes independent of study group allocation, skip to section 3.

For peer review only

	Descriptions as stated in the report/paper
**Randomisation and blinding:**	
**Sample size per group**	Intervention:<br>Control:
**Any indication for baseline differences between study groups?**	Yes/No/Unclear<br>Details:
**Comparison group description**	
**Intervention aim**	
**Is the explicit main aim of the intervention to increase patient activation or to target patients' knowledge, confidence and skills for self-management?**	*Yes/No/Unclear*<br>*(Delete as appropriate. Select No if the patient activation component forms part of a larger complex intervention).*
**Is patient activation the main component of the intervention?**	
**Intervention description**	
**Group or individual delivery**	
**Mode of delivery (e.g. web, face-to-face)**	
**Duration of intervention**	
**Timing (e.g. frequency, duration of each session)**	
**Providers (e.g. profession and training received)**	
**Intention to treat analysis?**	*Yes/No/Unclear (Delete as appropriate).*
**Any further notes:**	

Section 3: Outcomes & Measures

**PAct measure**	
**PAct measure used as continuous measure, ordinal (levels 1-4), or dichotomous (high/low)?**	Continuous/ordinal/dichotomous<br>(delete as appropriate)
**Time points measured/reported (for all outcomes):**	

For peer review only - http://bmjopen.bmj.com/site/about/guidelines.xhtml

**Covariates:** *(Note: Only extract covariates that were included in models that assessed the association between PAct and the outcomes of interest as per review protocol)*	

Clinical outcomes

Note: If outcomes not measured, please insert "n/a"

	**How measured/defined (+unit of measurement)**	**Source (e.g. self-report, health records)**
HbA1C level/glycaemic control		
Systolic blood pressure, diastolic blood pressure		
Low-density lipoprotein (LDL)<br>High-density lipoprotein (HDL)<br>Total cholesterol		
Serum triglycerides		
BMI		
weight		
Life expectancy/survival		

Self-management behaviours

Note: If outcomes not measured, please insert "n/a"

	**Self-report? (Yes/No/Unclear)**	**How defined/measured?** ***e.g. "consuming 5 servings of fruit/veg per day (Yes/No)"***
Diet		
Physical activity		
Smoking		
Alcohol consumption		
Medication adherence		

For peer review only - http://bmjopen.bmj.com/site/about/guidelines.xhtml

Section 4: Analyses + Results

Please extract data for adjusted and unadjusted associations (i.e. associations just between PAct and the relevant outcome [=unadjusted], and those where a model such as a linear regression is used to control for confounders [=adjusted]). If extracting data for both adjusted and unadjusted associations, please add additional rows to the table (e.g. an additional row labelled 'Cross-sectional association with PAct' so that you have one for the adjusted and one for the unadjusted data).

If several time points are reported, extract data for the longest follow-up time point.

If several variables were used for the same outcome please copy and paste the table and add details for the respective variable (for example, create a second table for "diet", and add the variable.

If the format of the tables is unsuitable for the reported results, please paste the relevant results into the 'other/comments' section.

How were missing data handled? *(e.g. multiple imputation)*	

**Outcome: HbA1c/glycaemic control**						
**How measured/defined:**						
	**Statistical test**	**Adjusted/ unadjusted?**	**Covariates (if adjusted)**	**Effect size for the association** (e.g. χ2, F, t or p values, Odds ratios, beta coefficients)	**p**	**Sample size**
Cross-sectional association with PAct:						
If intervention/longitudinal: Association between baseline PAct and subsequent outcome:						
If intervention/longitudinal: Association between baseline PAct and change in outcome:						
If intervention/longitudinal:						

For peer review only - http://bmjopen.bmj.com/site/about/guidelines.xhtml

Association between change in PAct and subsequent outcome:						
If intervention/longitudinal: Association between change in PAct and change in outcome:						
Other/comments:						

To extract data for further outcomes, please copy and paste the table above and edit the "outcome" field.

Outcomes:

- systolic blood pressure
- diastolic blood pressure
- LDL/HDL/Total cholesterol
- serum triglycerides
- weight
- BMI
- Life expectancy/survival
- Diet
- Physical activity
- Smoking
- Alcohol
- Medication adherence

Mediation:

Only if intervention study. Add details of any formal mediation analyses to determine if PAct mediates intervention effects on outcomes.

Section 5: Conclusions

**Conclusions**	
**Author's conclusions:**	

For peer review only - http://bmjopen.bmj.com/site/about/guidelines.xhtml

**Limitations (e.g. multiplicity)**	
**Reviewer's conclusions/comments:**	

For peer review only

Reporting checklist for protocol of a systematic review and meta analysis.

Based on the PRISMA-P guidelines.

Instructions to authors

Complete this checklist by entering the page numbers from your manuscript where readers will find each of the items listed below.

Your article may not currently address all the items on the checklist. Please modify your text to include the missing information. If you are certain that an item does not apply, please write "n/a" and provide a short explanation.

Upload your completed checklist as an extra file when you submit to a journal.

In your methods section, say that you used the PRISMA-Preporting guidelines, and cite them as:

Moher D, Shamseer L, Clarke M, Ghersi D, Liberati A, Petticrew M, Shekelle P, Stewart LA. Preferred Reporting Items for Systematic Review and Meta-Analysis Protocols (PRISMA-P) 2015 statement. Syst Rev. 2015;4(1):1.

		Reporting Item	Page Number
**Title**			
Identification	#1a	Identify the report as a protocol of a systematic review	1
Update	#1b	If the protocol is for an update of a previous systematic review, identify as such	n/a
**Registration**			
	#2	If registered, provide the name of the registry (such as PROSPERO) and registration number	2
**Authors**			
Contact	#3a	Provide name, institutional affiliation, e-mail address of all protocol authors; provide physical mailing address of corresponding author	1
Contribution	#3b	Describe contributions of protocol authors and identify the guarantor of the review	18

For peer review only

**Amendments**			
	#4	If the protocol represents an amendment of a previously completed or published protocol, identify as such and list changes; otherwise, state plan for documenting important protocol amendments	17
**Support**			
Sources	#5a	Indicate sources of financial or other support for the review	18
Sponsor	#5b	Provide name for the review funder and / or sponsor	18
Role of sponsor or funder	#5c	Describe roles of funder(s), sponsor(s), and / or institution(s), if any, in developing the protocol	18
**Introduction**			
Rationale	#6	Describe the rationale for the review in the context of what is already known	3-5
Objectives	#7	Provide an explicit statement of the question(s) the review will address with reference to participants, interventions, comparators, and outcomes (PICO)	5
**Methods**			
Eligibility criteria	#8	Specify the study characteristics (such as PICO, study design, setting, time frame) and report characteristics (such as years considered, language, publication status) to be used as criteria for eligibility for the review	6-8
Information sources	#9	Describe all intended information sources (such as electronic databases, contact with study authors, trial registers or other grey literature sources) with planned dates of coverage	8
Search strategy	#10	Present draft of search strategy to be used for at least one electronic database, including planned limits, such that it could be repeated	8-9
Study records - data management	#11a	Describe the mechanism(s) that will be used to manage records and data throughout the review	10
Study records - selection process	#11b	State the process that will be used for selecting studies (such as two independent reviewers) through each phase of the review (that is, screening, eligibility and inclusion in meta-analysis)	10
Study records - data	#11c	Describe planned method of extracting data from reports (such as	9

For peer review only

collection process		piloting forms, done independently, in duplicate), any processes for obtaining and confirming data from investigators	
Data items	#12	List and define all variables for which data will be sought (such as PICO items, funding sources), any pre-planned data assumptions and simplifications	10; 25
Outcomes and prioritization	#13	List and define all outcomes for which data will be sought, including prioritization of main and additional outcomes, with rationale	10; 25
Risk of bias in individual studies	#14	Describe anticipated methods for assessing risk of bias of individual studies, including whether this will be done at the outcome or study level, or both; state how this information will be used in data synthesis	10
Data synthesis	#15a	Describe criteria under which study data will be quantitatively synthesised	15
Data synthesis	#15b	If data are appropriate for quantitative synthesis, describe planned summary measures, methods of handling data and methods of combining data from studies, including any planned exploration of consistency (such as I2, Kendall's τ)	15-16
Data synthesis	#15c	Describe any proposed additional analyses (such as sensitivity or subgroup analyses, meta-regression)	16
Data synthesis	#15d	If quantitative synthesis is not appropriate, describe the type of summary planned	14
Meta-bias(es)	#16	Specify any planned assessment of meta-bias(es) (such as publication bias across studies, selective reporting within studies)	16
Confidence in cumulative evidence	#17	Describe how the strength of the body of evidence will be assessed (such as GRADE)	11-14

The PRISMA-P elaboration and explanation paper is distributed under the terms of the Creative Commons Attribution License CC-BY. This checklist was completed on 07. August 2021 using https://www.goodreports.org/, a tool made by the EQUATOR Network in collaboration with Penelope.ai

For peer review only
